# Isobavachalcone attenuates experimental periodontitis by suppressing PTGS2-associated macrophage ferroptosis and restoring periodontal immune homeostasis

**DOI:** 10.3389/fphar.2026.1913548

**Published:** 2026-09-21

**Authors:** Ruolan Yang, Yanye Jin, Ziyi Wang, Zeyan Peng, Liang Yang, Zhi Qi, Jing Shen

**Affiliations:** 1 Department of International VIP Dental Clinic, Tianjin Stomatological Hospital, School of Medicine, Nankai University, Tianjin, China; 2 Tianjin Key Laboratory of Oral and Maxillofacial Function Reconstruction and Stomatology Institute of Nankai University, Tianjin, China; 3 Tianjin Medical University, Tianjin, China; 4 Department of Molecular Pharmacology, School of Medicine, Nankai University, Tianjin, China; 5 Department of General Surgery, Tianjin Union Medical Center, The First Affiliated Hospital of Nankai University, Tianjin, China

**Keywords:** ferroptosis, isobavachalcone, macrophage polarization, periodontitis, PTGS2

## Abstract

**Background:**

Periodontitis (PD) is a destructive inflammatory disease driven by the collapse of immune homeostasis and alveolar bone resorption. Isobavachalcone (IBC) is a naturally occurring prenylated chalcone belonging to the plant flavonoid family, mainly enriched in the mature seeds of *Cullen corylifolium*, with reported anti-inflammatory, antioxidant, and osteoprotective activities. However, the molecular mechanisms by which IBC modulates the macrophage-mediated immune response and cellular survival within the chronic inflammatory microenvironment of periodontitis remain poorly understood. This study aimed to investigate the therapeutic efficacy of IBC on periodontitis and elucidate its potential molecular targets, with a specific focus on macrophage ferroptosis and immunometabolic regulation.

**Methods:**

Experimental periodontitis was induced in rats by ligation combined with Porphyromonas gingivalis LPS topical application. Alveolar bone parameters were analyzed via micro-CT and histology. Network pharmacology, molecular dynamics (MD) simulations, cellular thermal shift assays (CETSA) and microscale thermophoresis (MST) assays were integrated to screen and validate the physical binding targets of IBC. Single-cell RNA sequencing (scRNA-seq) analysis and clinical samples were utilized to confirm translational relevance, and *in vitro* RAW264.7 macrophage models were employed for mechanistic verification.

**Results:**

*In vivo*, subgingival administration of IBC significantly reduced alveolar bone loss, suppressed inflammatory infiltration, and promoted macrophage M2 polarization. Bioinformatic screening combined with MD simulations and CETSA validated PTGS2 as the direct functional target of IBC. Human scRNA-seq and clinical specimen analyses confirmed that PTGS2 expression is predominantly enriched in periodontal macrophages and inversely correlates with the expression of glutathione peroxidase 4 (GPX4). Mechanistically, IBC physically bound and antagonized PTGS2, thereby blocking the downstream JNK/p38 MAPK phosphorylation cascade and breaking the noxious reactive oxygen species (ROS) amplification loop. Consequently, IBC restored mitochondrial iron transport homeostasis (downregulating TFR1/MFRN1 and upregulating CISD1), stabilized GPX4, and prevented macrophage ferroptosis, which collectively drove the transition to a reparative M2 phenotype. Crucially, genetic overexpression of PTGS2 abolished these protective effects against ferroptosis *in vitro*.

**Conclusion:**

IBC acts as a potent protective agent against periodontitis by directly targeting the PTGS2–MAPK–ROS–GPX4 axis, thereby inhibiting macrophage ferroptosis and restoring periodontal immune homeostasis.

## Introduction

Periodontitis (PD) is defined as a chronic inflammatory disease of the tooth-supporting tissues initiated by dysbiotic dental plaque and sustained by an imbalanced host immune response (Tonetti et al., 2017; Diseases and Injuries, 2020; Wu et al., 2025; Zhang et al., 2026). Its principal pathological hallmarks include gingival inflammation, periodontal attachment loss, progressive alveolar bone resorption, and, ultimately, tooth loosening and tooth loss (Pihlstrom et al., 2005; Papapanou et al., 2018; Wang et al., 2025). Under physiological conditions, the periodontal immune response contributes to host defense by limiting microbial invasion, clearing pathogenic stimuli, and maintaining tissue homeostasis. However, when microbial challenge persists or the inflammatory response becomes excessive, this protective immune reaction turns pathogenic, leading to continuous recruitment and activation of immune cells, uncontrolled production of inflammatory mediators, oxidative stress, collagen degradation, and osteoclast-driven bone destruction (Peres et al., 2019; Nascimento et al., 2024; Collaborators, 2025). This transition from host defense to immune-mediated tissue injury forms a vicious cycle of “microbial dysbiosis–inflammation–bone loss”, which is a central mechanism underlying the progression and recurrence of PD (Hajishengallis, 2014; Van Dyke and Sima, 2020). Clinically, mechanical debridement and adjunctive antimicrobial therapy remain the mainstay of periodontal treatment; nevertheless, these approaches primarily reduce the microbial burden and do not fully restore the disrupted immune microenvironment. As a result, PD remains prone to recurrence and progression, representing a common and intractable inflammatory disease that severely compromises masticatory function, tooth retention, and quality of life (Sanz et al., 2020).

Macrophages are key innate immune cells within periodontal tissues and act as central regulators of host defense, inflammatory resolution, and tissue remodeling during periodontitis (Sun et al., 2021). Under homeostatic conditions, macrophages continuously monitor the local microenvironment, phagocytose pathogenic bacteria and apoptotic cells, and secrete regulatory mediators to maintain periodontal immune balance. However, upon prolonged exposure to pathogen-associated molecular patterns, inflammatory cytokines, and oxidative stress, macrophages undergo phenotypic polarization, which is commonly characterized by a pro-inflammatory M1-like state and a reparative or anti-inflammatory M2-like state (Lamont et al., 2018). M1-like macrophages are typically driven by Toll-like receptor activation and interferon-related signaling, producing high levels of pro-inflammatory mediators such as iNOS, TNF-α, IL-1β, and IL-6, thereby sustaining tissue inflammation, promoting osteoclast activation, and impairing periodontal repair. In contrast, M2-like macrophages contribute to inflammation resolution and tissue reconstruction by secreting anti-inflammatory cytokines and pro-reparative factors, including IL-10, Arg1, and CD206 (Zhang et al., 2021; Shi et al., 2024). During periodontitis, persistent microbial stimulation and uncontrolled reactive oxygen species (ROS) accumulation favor M1-dominant macrophage activation while limiting M2-associated reparative responses, resulting in unresolved inflammation and progressive alveolar bone destruction. Accordingly, restoring the balance between M1-like inflammatory activation and M2-like reparative polarization has emerged as an important immunopharmacological strategy for controlling periodontal inflammation and promoting tissue homeostasis (Han et al., 2022; Gonzalez et al., 2023).

In recent years, ferroptosis—a regulated form of programmed cell death (PCD) driven by lipid peroxidation and dependent on iron—has been identified as a key pathological mechanism in inflammatory diseases (Chen et al., 2024; Jin et al., 2024). Upon stimulation by damage-associated molecular patterns (DAMPs), such as bacterial lipopolysaccharides, the excessive production of ROS synergizes with iron overload to induce ferroptosis, thereby triggering persistent inflammation and tissue damage (Shen et al., 2025). Specifically, emerging evidence highlights the pivotal role of ferroptosis in periodontitis-induced alveolar bone loss. Recent studies have demonstrated that periodontal pathogens, such as *Porphyromonas gingivalis*, can trigger ferroptosis in macrophages, thereby escalating the release of pro-inflammatory cytokines and directly modulating osteoclast differentiation (Qiao et al., 2023; Bao et al., 2024). This process is characterized by the downregulation of Glutathione Peroxidase 4 (GPX4) and a subsequent rise in lipid peroxides, which further facilitates the recruitment of osteoclasts and aggravates alveolar tissue destruction (Chen K. et al., 2022). However, the upstream molecular regulatory mechanisms linking inflammation, ROS amplification, and ferroptosis within macrophages during the pathogenesis of periodontitis remain to be fully elucidated (Jiang et al., 2025).

Isobavachalcone (IBC) is a naturally occurring prenylated chalcone belonging to the plant flavonoid family. It is mainly enriched in the mature seeds of *Cullen corylifolium* (previously referred to as *Psoralea corylifolia* L.) and is also distributed in several medicinal plants from the Fabaceae and Moraceae families (China, 2020; Chen et al., 2023). As a structurally defined small molecule, IBC has attracted increasing attention because of its broad pharmacological properties, including anti-inflammatory, antioxidant, antibacterial, neuroprotective, and osteoprotective activities (Wang M. et al., 2021; Xing et al., 2022). Previous studies have shown that IBC can ameliorate inflammatory bowel injury by suppressing NF-κB p65 signaling, regulate inflammatory activation and cell survival in rheumatoid arthritis models through PI3K/AKT and JAK/STAT-related pathways, and inhibit bone loss by suppressing ERK/NF-κB signaling and M1 macrophage polarization (Wang X. et al., 2021; Zhou et al., 2021a; Liu et al., 2022; Wang et al., 2022). More recently, IBC has also been reported to alleviate neuroinflammatory pathology by promoting autophagy-mediated amyloid-βclearance and inhibiting NLRP3 inflammasome activation, further supporting its potential as an immunomodulatory natural small molecule (Kour et al., 2025). Although macrophage dysregulation and ferroptosis have been implicated in the progression of periodontitis, whether IBC can restore periodontal redox homeostasis, facilitate tissue repair, and rebalance macrophage M1/M2 polarization by suppressing macrophage ferroptosis remains unknown. The molecular mechanisms underlying these effects also require further clarification.

Therefore, this study aimed to investigate the protective effects of IBC against experimental periodontitis, elucidate its underlying mechanism in regulating macrophage ferroptosis and immune polarization, and provide a pharmacological basis for targeting host immune dysregulation in periodontal inflammatory destruction.

## Materials and methods

### Chemicals and reagents

Isobavachalcone (IBC, purity ≥98%) was purchased from MedChemExpress (Monmouth Junction, NJ, United States of America). To ensure the authenticity and chemical reliability of this commercially sourced compound, liquid chromatography-mass spectrometry (LC-MS) chromatograms and the certificate of analysis provided by the manufacturer are presented in Supplementary Figure S1. IBC powder was dissolved in dimethyl sulfoxide (DMSO; Sigma-Aldrich, St. Louis, MO, United States of America) and stored at −20 °C in the dark. SP600125 (a selective JNK inhibitor, purity ≥99%) and SB203580 (a selective p38 inhibitor, purity ≥99%) were purchased from MedChemExpress (Monmouth Junction, NJ, United States of America). Both inhibitors were dissolved in dimethyl sulfoxide (DMSO; Sigma-Aldrich, St. Louis, MO, United States of America) to prepare stock solutions and stored at −20 °C in the dark until use. Celecoxib was purchased from MedChemExpress (Monmouth Junction, NJ, United States of America), dissolved in DMSO, and stored at −20 °C until use.

Porphyromonas gingivalis lipopolysaccharide (Pg-LPS) was obtained from InvivoGen (San Diego, CA, United States of America). RSL3 and N-acetylcysteine (NAC) were purchased from Selleck Chemicals (Houston, TX, United States of America). Cell Counting Kit-8 (CCK-8), Calcein-AM/PI staining kit, MitoSOX Red, tetramethylrhodamine ethyl ester (TMRE), and lipid peroxidation detection kits were obtained from Beyotime Biotechnology (Shanghai, China).

Primary antibodies used for Western blotting and immunofluorescence analyses, including their sources, catalog numbers, and dilution ratios, are summarized in Supplementary Table S1. Antibodies against oxidative stress markers (8-OHdG and 4-HNE) and ELISA kits for cytokine detection were obtained from Elabscience Biotechnology (Wuhan, China). All other reagents were of analytical grade.

## Experimental periodontitis model and treatment protocol

All animal experiments were approved by the Institutional Animal Care and Use Committee of Tianjin Stomatological Hospital (Approval No. PA2025-B-002). All animal experiments were conducted in accordance with the ARRIVE guidelines and the United Kingdom. Animals (Scientific Procedures) Act, 1986 and associated guidelines. Male Sprague-Dawley rats (6–8 weeks old, 200–220 g) were purchased from SPF Biotechnology Co., Ltd. (Beijing, China) and housed under standard laboratory conditions (12-h light/dark cycle, controlled temperature and humidity). We randomly assigned thirty rats into the following five groups: (1) control group; (2) periodontitis model group; (3) IBC low-dose group (2 mg/mL); (4) IBC high-dose group (5 mg/mL); and (5) minocycline (2 mg/mL) treatment group (positive control).

To establish the experimental periodontitis model, we used a ligature-associated bacterial stimulation protocol (Lin et al., 2021; Ai et al., 2022). Briefly, under isoflurane anesthesia, a 4–0 orthodontic ligature wire was bilaterally placed around the maxillary second M. Subsequently, Pg-LPS (1 mg/mL, 10 μL) was topically applied into the gingival sulcus every other day to maintain bacterial inflammatory stimulation. The ligatures were continuously maintained throughout the 14-day induction period.

Following the completion of the treatment period, animals were deeply anesthetized by intraperitoneal injection of 10% chloral hydrate (3 mL/kg). After confirming the loss of reflexes and achieving a surgical plane of anesthesia, animals were euthanized, and maxillary bone and gingival tissues were collected for subsequent micro-CT, histological, immunofluorescence, and molecular analyses.

The dosages of IBC were primarily determined based on our preliminary dose-finding experiments. Due to the anatomical constraints of the thin rat gingival tissue, the injection volume was strictly limited to 10 μL per site. Based on the administered concentration and injection volume, the actual doses of IBC were calculated as 20 μg/site for the low-dose group (2 mg/mL × 10 μL) and 50 μg/site for the high-dose group (5 mg/mL × 10 μL). Our preliminary results demonstrated that 5 mg/mL (50 μg/site) was the maximum safe dose that effectively reduced inflammatory infiltration without inducing localized gingival erythema or tissue necrosis; therefore, it was selected as the high-dose treatment, while 2 mg/mL (20 μg/site) was selected as the low-dose treatment. These concentrations also aligned with the effective range of topical chalcone administration reported in previous experimental periodontitis model (Frazão et al., 2024). Treatment commenced on day 8 after periodontitis induction. IBC or minocycline was administered via subgingival injection (10 μL per site) every 3 days until day 28. Following euthanasia, maxillary bone and gingival tissues were harvested for subsequent analyses.

### Micro-computed tomography (Micro-CT) analysis

Maxillary specimens were fixed in 4% paraformaldehyde (Solarbio, Beijing, China) for 48 h and scanned using a high-resolution micro-CT system (VNC-102, Pingseng Healthcare Technology, Beijing, China). Three-dimensional (3D) reconstruction was performed using the manufacturer’s software. Alveolar bone loss was quantified by measuring the distance from the cementoenamel junction (CEJ) to the alveolar bone crest (ABC) at standardized sites around the maxillary second M. Furthermore, to evaluate the microarchitecture of the alveolar bone, a volume of interest (VOI) was defined within the interradicular bone of the second M to calculate the following morphometric parameters: bone volume fraction (BV/TV), trabecular thickness (Tb.Th), trabecular number (Tb.N), and trabecular separation (Tb.Sp). Investigators responsible for micro-CT reconstruction, histological evaluation, and quantitative fluorescence analysis were blinded to the experimental group allocation during data analysis.

### Histological, immunohistochemical, and immunofluorescence analyses

Fixed maxillae were decalcified in 10% EDTA (Servicebio, Wuhan, China) for 4 weeks, dehydrated, and embedded in paraffin. Sagittal sections (4–5 μm) were prepared.

Hematoxylin–eosin (H&E) staining was used to evaluate inflammatory infiltration, and Masson’s trichrome staining (Solarbio, Beijing, China) was performed to assess collagen degradation. Tartrate-resistant acid phosphatase (TRAP) staining was conducted using a commercial kit (Servicebio, Wuhan, China) to identify osteoclast activity. For immunohistochemistry (IHC), sections were incubated with anti-PTGS2 antibody (1:200) at 4 °C overnight, followed by HRP-conjugated secondary antibody incubation and DAB visualization (ZSGB-BIO, Beijing, China). Immunofluorescence staining was performed to evaluate macrophage polarization and specific protein expression. Briefly, following deparaffinization, rehydration, and antigen retrieval (using citrate buffer, pH 6.0), the sections were permeabilized and blocked with 5% bovine serum albumin (BSA) containing 0.3% Triton X-100 for 1 h at room temperature. Subsequently, the sections were incubated overnight at 4 °C with primary antibodies diluted according to the manufacturer’s instructions. After washing with PBS, the slides were incubated for 1 h at room temperature in the dark with corresponding fluorophore-conjugated secondary antibodies (Abcam, Cambridge, United Kingdom): anti-PTGS2, anti-GPX4, anti-iNOS, and anti-CD206 were labeled with a red fluorescent secondary antibody, whereas anti-CD68 was labeled with a green fluorescent secondary antibody. Nuclei were counterstained with DAPI for 5 min. Finally, the slides were mounted with an anti-fade mounting medium, and images were captured using a fluorescence microscope (Olympus BX53, Olympus Corporation, Tokyo, Japan). The fluorescence intensity and positive cell ratios were quantitatively analyzed using ImageJ software (National Institutes of Health, Bethesda, MD, United States of America).

### Network pharmacology and target prediction

The potential pharmacological targets of IBC were identified using multiple complementary databases, including SwissTargetPrediction, Similarity Ensemble Approach (SEA), HERB 2.0, TCMBank, and the Comparative Toxicogenomics Database (CTD). Periodontitis-associated genes were collected from GeneCards, DisGeNET, DrugBank, Online Mendelian Inheritance in Man (OMIM), and the Therapeutic Target Database (TTD). After removal of duplicated entries, the predicted IBC targets and disease-associated genes were intersected to identify potential therapeutic targets.

Overlapping targets were visualized using Venn diagrams. Protein–protein interaction (PPI) networks were constructed using the STRING database with a highest confidence score (>0.9) to ensure the reliability of the interactions. The network was then visualized in Cytoscape (version 3.9.1). Hub genes were identified using three complementary network topology algorithms, including MCC analysis in cytoHubba, MCODE clustering analysis, and cytoNCA analysis based on degree and betweenness centrality. Candidate hub genes identified by these independent algorithms were further intersected to determine the core targets.

### GO, KEGG, and GSEA analyses

Gene Ontology (GO) and Kyoto Encyclopedia of Genes and Genomes (KEGG) enrichment analyses were performed using the SRplot online platform (Tang et al., 2023; https://www.bioinformatics.com.cn, accessed on 10 December 2024). Results were visualized as bubble and bar plots.

Gene Set Enrichment Analysis (GSEA) was conducted using dataset GSE10334, which was selected because it provides human gingival transcriptomic profiles from periodontitis patients and healthy controls with sufficient sample size and robust differential expression information, enabling evaluation of disease-associated biological pathways. Background gene sets were obtained from the MSigDB database (HALLMARK and WikiPathways). A false discovery rate (FDR) < 0.05 and P < 0.05 were considered statistically significant.

### Molecular docking

Molecular docking was performed using Molecular Operating Environment (MOE) 2019 (Chemical Computing Group, Montreal, Canada) and AutoDock Vina (version 1.1.2). The three-dimensional structure of PTGS2 was obtained from the RCSB Protein Data Bank (PDB), and the chemical structure of IBC was retrieved from PubChem. Before docking, the protein structure was prepared using PyMOL (version 3.0.3) and AutoDockTools (version 1.5.6). Briefly, the protein structure was subjected to preprocessing procedures, including removal of crystallographic water molecules and unnecessary small molecules, addition of hydrogen atoms, correction of atom types and partial charges, and structural optimization to obtain a suitable receptor model for docking analysis. The ligand structure of IBC was similarly prepared by adding hydrogen atoms and assigning appropriate atomic parameters before docking.

During docking, 50 candidate conformations were generated using the MOE conformational sampling algorithm. These conformations were ranked according to the MOE scoring function, and the top 10 conformations with the highest docking scores were further evaluated based on binding affinity, spatial orientation, and interaction patterns with the target protein. The optimal docking pose was selected as the representative binding model and subsequently used as the initial structure for molecular dynamics simulation analysis. Binding affinity values (kcal/mol) and detailed interaction characteristics were analyzed to evaluate the potential interaction between IBC and PTGS2.

### Molecular dynamics simulations

Molecular dynamics (MD) simulations were performed using GROMACS (version 2023) to evaluate the stability and dynamic characteristics of the IBC–PTGS2 complex. The optimal docking conformation obtained from MOE 2019 was selected as the initial structure for MD simulation. Before simulation, the protein–ligand complex was subjected to structural preparation. The protein was parameterized using the AMBER99SB-ILDN force field, while the ligand IBC was parameterized using the General Amber Force Field (GAFF).

The complex was solvated in a cubic water box (10 × 10 × 10 nm^3^) using the TIP3P water model, with a minimum distance of 1.2 nm between the protein surface and the boundary of the simulation box. Sodium and chloride ions (Na^+^/Cl^−^) were added to neutralize the system. Energy minimization was subsequently performed using the steepest descent algorithm with a maximum of 50,000 steps to remove unfavorable contacts.

After energy minimization, the system was equilibrated under the NVT and NPT ensembles. The temperature was maintained at 300 K using the V-rescale thermostat, while the pressure was maintained at 1 bar using the Berendsen barostat. Long-range electrostatic interactions were calculated using the Particle Mesh Ewald (PME) method, and the cutoff distances for Coulombic and van der Waals interactions were both set to 1.0 nm (10 Å).

Following equilibration, a 100-ns production simulation was conducted. The stability and dynamic behavior of the IBC–PTGS2 complex were evaluated by trajectory analyses, including root mean square deviation (RMSD), root mean square fluctuation (RMSF), radius of gyration (Rg), intermolecular hydrogen bond analysis, free energy landscape (FEL) analysis, and MM/GBSA binding free energy calculations.

### Cellular thermal shift assay (CETSA)

To experimentally validate the physical interaction between IBC and its potential target, CETSA was performed using RAW 264.7 macrophages. Cells were treated with DMSO or 36 μM IBC for 2 h at 37 °C. The harvested cells were resuspended in PBS supplemented with protease inhibitors and distributed into PCR tubes. Each aliquot was heated at a specific temperature gradient (37, 40, 43, 46, 49, 52, 55, 58, 61, and 64 °C) for 3 min using a thermal cycler, followed by incubation at room temperature for 3 min. The cells were then lysed by three rapid freeze-thaw cycles using liquid nitrogen. The soluble protein fractions were isolated by centrifugation at 12,000 × *g* for 20 min at 4 °C. The thermal stability of PTGS2 (74 kDa) was analyzed by Western blotting, with GAPDH (36 kDa) serving as a non-thermally sensitive loading control. The relative band intensities were quantified using ImageJ software, and melting curves were generated to compare the thermal stability shift between the control and IBC-treated groups.

### Microscale thermophoresis (MST) assay

Recombinant human PTGS2 protein was expressed in *E. coli* and purified by Ni-NTA affinity chromatography according to the manufacturer’s instructions. The purified protein was labeled with RED-tris-NTA dye (NanoTemper Technologies) following the standard labeling protocol. Unreacted dye was removed by buffer exchange using a desalting column.

For MST measurements, the labeled PTGS2 protein was diluted to a final concentration of approximately 20 nM in MST buffer (20 mM Tris-HCl, pH 7.4, 150 mM NaCl, 0.05% Tween-20). IBC was dissolved in DMSO and serially diluted to obtain a concentration series ranging from 0 to 5 μM, with a constant final DMSO concentration of 5%. Each IBC dilution was mixed with an equal volume of labeled PTGS2 and incubated for 15–30 min at room temperature in the dark. The samples were then loaded into standard Monolith NT.115 capillaries, and MST traces were recorded at 25 °C using 20%–40% MST power and 60%–80% LED power.

All measurements were performed as three independent biological replicates. The normalized fluorescence (Fnorm) values were plotted against the logarithm of IBC concentrations, and the dissociation constant (Kd) was calculated by fitting the dose–response curves using the MO. Affinity Analysis software (NanoTemper Technologies) with the Kd model. Data are presented as mean ± SD from three independent experiments.

#### COX-2/PTGS2 activity assay

RAW264.7 cells were seeded into 6-well plates and divided into four groups: control, LPS, LPS + IBC (36 μM), and LPS + celecoxib (36 μM). After the indicated treatments, cells were collected and lysed. The cell lysates were centrifuged, and the supernatant was collected for the measurement of COX activity. COX enzymatic activity was determined using a COX activity fluorescence assay kit (Solarbio, Cat. No. BC662F) according to the manufacturer’s protocol. Fluorescence intensity was measured using a fluorescence microplate reader at the recommended excitation and emission wavelengths. All experiments were performed in triplicate as three independent biological replicates. Data are presented as mean ± SD from three independent experiments. Statistical analysis was performed using one-way ANOVA followed by appropriate *post hoc* tests.

### scRNA-seq data analysis

The scRNA-seq dataset (GSE171213) was retrieved from the Gene Expression Omnibus (GEO) database. This dataset was selected because it contains single-cell transcriptomic profiles of human periodontal gingival tissues with adequate sample size and high cellular resolution, allowing precise characterization of PTGS2 expression across different periodontal cell populations. Data processing was performed using the Seurat R package (version 4.0). Cells with fewer than 200 detected genes or mitochondrial gene proportions exceeding 10% were removed during quality control. After normalization and identification of highly variable genes, PCA was performed for dimensionality reduction, followed by UMAP visualization and clustering analysis.

Cell clusters were annotated according to canonical marker genes of major periodontal cell populations, including macrophages, fibroblasts, epithelial cells, and other immune populations. Differential expression analysis, violin plots, feature plots, and heatmaps were generated to characterize the expression patterns and cellular distribution of candidate genes across different clusters.

### Human clinical sample collection

This study was approved by the Ethics Committee of Tianjin Stomatological Hospital (Approval No. PH2026-W-001–001) and conducted in accordance with the Declaration of Helsinki. Gingival tissues were collected from periodontitis patients and healthy controls. Inclusion and exclusion criteria were defined according to the 2018 classification of periodontal diseases (Tonetti et al., 2018). Tissue samples (n = 15 per group) were fixed in 4% paraformaldehyde, embedded in paraffin, and sectioned (4 μm) for histological and molecular analyses.

### Cell culture

The RAW264.7 murine macrophage cell line was obtained from the American Type Culture Collection (ATCC, Manassas, VA, United States of America). The cells were maintained in Dulbecco’s Modified Eagle Medium (DMEM; Gibco, Grand Island, NY, United States of America) supplemented with 10% fetal bovine serum (FBS; Gibco, United States of America) and 1% penicillin-streptomycin (Sigma-Aldrich, St. Louis, MO, United States of America). The cultures were incubated in a humidified atmosphere containing 5% CO_2_ at 37 °C. The medium was refreshed every 2–3 days, and cells were passaged upon reaching 80% confluence using a cell scraper to ensure high viability and functional integrity for subsequent experiments.

### Plasmids and transfection

The PTGS2 overexpression plasmid (OE-PTGS2) and its corresponding negative control (NC) vector were purchased from Miaoling Bioscience (Wuhan, China). For transfection, RAW264.7 cells were seeded in culture plates and grown to 60%–70% confluence. Transient transfection was executed using Lipofectamine 3000 reagent (Invitrogen, Carlsbad, CA, United States of America) according to the manufacturer’s instructions. Briefly, the plasmid DNA and Lipofectamine 3000 were separately diluted in Opti-MEM (Gibco, United States of America), mixed to form liposome-plasmid complexes, and then added to the cell culture. After 6 h of incubation, the transfection medium was replaced with fresh complete DMEM to allow the cells to recover and the target protein to be successfully expressed.

### Cell grouping and treatments

To evaluate the preventive effects of IBC on the inflammatory response, RAW264.7 cells were assigned into various experimental groups. Following the transfection recovery period, cells were pre-incubated with indicated concentrations of IBC for 2 h. Subsequently, an *in vitro* inflammatory model was established by stimulating the cells with 1 μg/mL Lipopolysaccharide (LPS; Sigma-Aldrich, United States of America) for 48 h. To further investigate the mechanisms involving ferroptosis and oxidative stress, the ferroptosis inducer RSL3 (Selleck Chemicals, Houston, TX, United States of America) was administered to specifically targeted groups during the final phase of treatment, while the antioxidant N-acetylcysteine (NAC; Sigma-Aldrich, United States of America) was utilized as a mechanistic probe. After the 48-h treatment period, cells and culture supernatants were collected for further biochemical and molecular analysis.

### Mitochondrial ROS and membrane potential (MMP) analysis

Mitochondrial ROS levels were detected using the MitoSOX Red mitochondrial superoxide indicator (Beyotime, Shanghai, China). Briefly, RAW 264.7 cells were incubated with 2 μM MitoSOX Red at 37 °C for 30 min in the dark. After washing three times with warm PBS, the cells were imaged using a fluorescence microscope (Leica Microsystems, Wetzlar, Germany). The mean fluorescence intensity was quantified using ImageJ software to assess mitochondrial oxidative stress. For MMP assessment, cells were stained with TMRE (200 nM; Beyotime) for 20 min at 37 °C, according to the manufacturer’s instructions. The fluorescence images were captured immediately, and the red fluorescence intensity was analyzed to evaluate changes in mitochondrial polarization. Mitochondrial membrane potential was assessed using TMRE (200 nM).

### Lipid peroxidation and cytokine assay

Lipid peroxidation was evaluated by measuring malondialdehyde (MDA) levels using a thiobarbituric acid (TBA)-based assay kit (Beyotime, Shanghai, China). Total and oxidized glutathione (GSH and GSSG) levels in cell lysates were quantified using a commercial assay kit (Beyotime). For cytokine analysis, the concentrations of IL-1β, TNF-α, IL-6, and IL-10 in cell culture supernatants or serum were measured using specific ELISA kits (Elabscience, Wuhan, China). The absorbance at 450 nm was recorded using a microplate reader (Thermo Fisher Scientific, Waltham, MA, United States of America), and the final concentrations were calculated based on corresponding standard curves.

### Quantitative real-time PCR (qRT-PCR)

Total RNA was extracted using TRIzol reagent (Invitrogen, United States of America) and reverse-transcribed using a cDNA synthesis kit (Yeasen Biotechnology, Shanghai, China). qRT-PCR was performed using SYBR Green Master Mix (Roche, Basel, Switzerland). Gene expression levels were calculated using the 2^^−ΔΔCt^ method and normalized to GAPDH. Primer sequences used for quantitative real-time PCR are listed in Supplementary Table S2.

### Western blot analysis

Protein concentration was determined using a BCA protein assay kit (Thermo Fisher Scientific, Waltham, MA, United States of America). Equal amounts of protein (30 μg) were separated by SDS-PAGE and transferred to PVDF membranes (Millipore, United States of America). After blocking, membranes were incubated with primary antibodies overnight at 4 °C, followed by HRP-conjugated secondary antibodies. Signals were detected using an ECL kit (Thermo Fisher Scientific) and quantified using ImageJ. Uncropped Western blot images corresponding to the main figures are provided in Supplementary Figure S7.

### Statistical analysis

Data are presented as mean ± standard deviation (SD). All experiments were performed using independent biological replicates, and the exact sample sizes are indicated in the corresponding figure legends. Data distribution was assessed using the Shapiro–Wilk test before statistical analysis. Statistical analyses were performed using GraphPad Prism (version 9.5; GraphPad Software, San Diego, CA, United States of America). Comparisons between two groups were performed using an unpaired Student’s t-test, whereas comparisons among multiple groups were conducted using one-way analysis of variance (ANOVA) followed by Tukey’s *post hoc* test. For omics-related analyses, including transcriptomic and enrichment analyses, false discovery rate (FDR) correction was applied for multiple testing adjustment where appropriate. A two-sided P value <0.05 was considered statistically significant.

## Results

### IBC mitigates alveolar bone loss and inflammatory destruction in experimental PD rat model

Before evaluating the *in vivo* and *in vitro* pharmacological efficacy of IBC, we first characterized its chemical structure and rigorously verified the identity and purity of the commercially obtained compound. As depicted in Figure 1A, IBC is a naturally occurring prenylated chalcone featuring specific hydroxyl configurations. High-performance liquid chromatography (HPLC) analysis revealed a single predominant peak at a retention time of 1.24 min, confirming an ultra-high relative purity of 98.99% based on the peak area integration (Figures 1B,C). Furthermore, electrospray ionization mass spectrometry (ESI-MS) in positive ion mode displayed a prominent molecular ion peak at *m/z* 325.1 *[M + H]* ^*+*^ (Figure 1D), which perfectly aligns with the theoretical molecular weight of IBC. This definitive structural confirmation and exceptional chemical purity provide a highly reliable material basis for our subsequent biological experiments. We then established an experimental periodontitis rat model using ligature combined with LPS injection. The schematic timeline of the animal experimental design is illustrated in Figure 2A. The macroscopic establishment of the periodontitis model and the subsequent *in situ* subgingival delivery of therapeutic agents via micro-syringe are illustrated in Figure 2B. Three-dimensional and sectional Micro-CT images revealed severe alveolar bone destruction in the PD group (Figure 2C). Specifically, the buccal side view and coronal planes showed significant bone resorption on both buccal and lingual sides, while the sagittal planes demonstrated extensive bone loss at the mesial and distal surfaces. Conversely, IBC treatment markedly preserved the bone architecture, with the IBC-High group maintaining a significantly more intact alveolar bone crest and reduced root exposure compared to the untreated PD group. Quantitative parameters further confirmed the osteoprotective effects of IBC (Figures 2D–G). Compared with the Control group, PD rats showed a dramatic decrease in Tb.N and BS/TV, while Tb. Sp and CEJ-ABC distance were significantly increased (P < 0.0001). However, IBC administration (especially at high doses) effectively reversed these trends. Specifically, the IBC-High group demonstrated significant improvements in trabecular micro-architecture and a marked reduction in CEJ-ABC distance, showing comparable efficacy to the Minocycline positive control. The results of H&E and Masson’s trichrome staining showed that IBC significantly reduced the number of inflammatory cells and promoted the repair of collagen fibers (Figure 2H). Furthermore, TRAP staining demonstrated a dramatic decrease in osteoclast numbers upon IBC treatment. Consistent with these phenotypic observations, the local production of pro-inflammatory cytokines (TNF-α, IL-6, IL-1β) was notably suppressed (Figures 2I–M). qRT-PCR analysis further confirmed that IBC dose-dependently rescued the osteogenic/osteoclastogenic marker balance (upregulating *Runx2, Opn, Alp* and downregulating *Ctsk, Trap, Nfatc1*) *in vivo* (Figures 2M–P; Supplementary Figure S1A-F).

**FIGURE 1:**
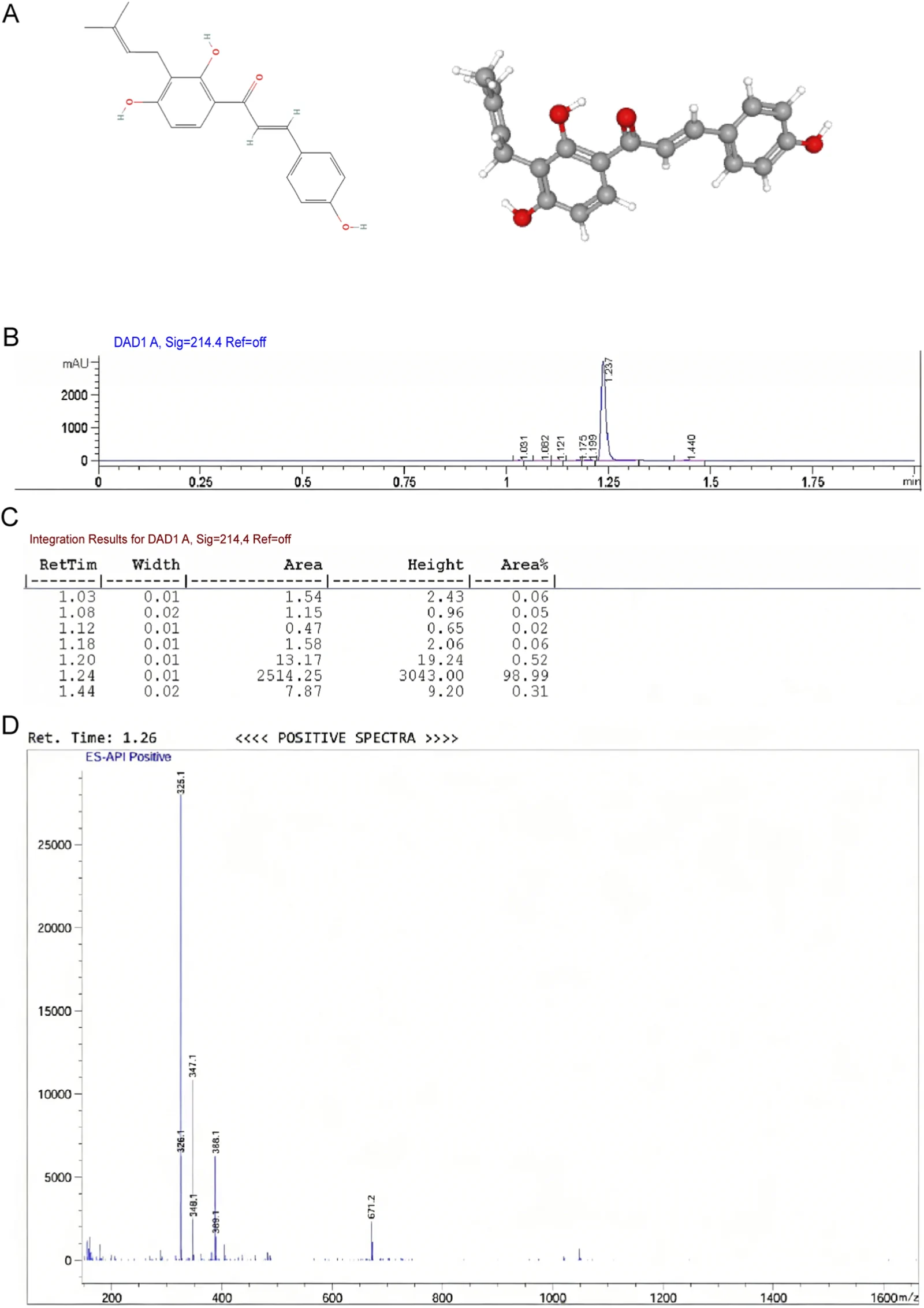
Purity validation and mass spectrometry characterization of Isobavachalcone (IBC). **(A)** 2D and 3D chemical structures of Isobavachalcone (IBC). **(B)** Ultra-performance liquid chromatography (UPLC) chromatogram of the commercially procured IBC. The major peak observed at a retention time of 1.24 min represents the target compound. **(C)** Integration results of the DAD signal at 214 nm, demonstrating a relative purity of 98.99% based on the peak area percentage. **(D)** Mass spectrometry (MS) analysis of IBC in electrospray ionization positive (ESI+) mode. The prominent molecular ion peak at *m/z* 325.1 *[M + H]* ^*+*^ confirms the theoretical molecular weight of IBC.

**FIGURE 2:**
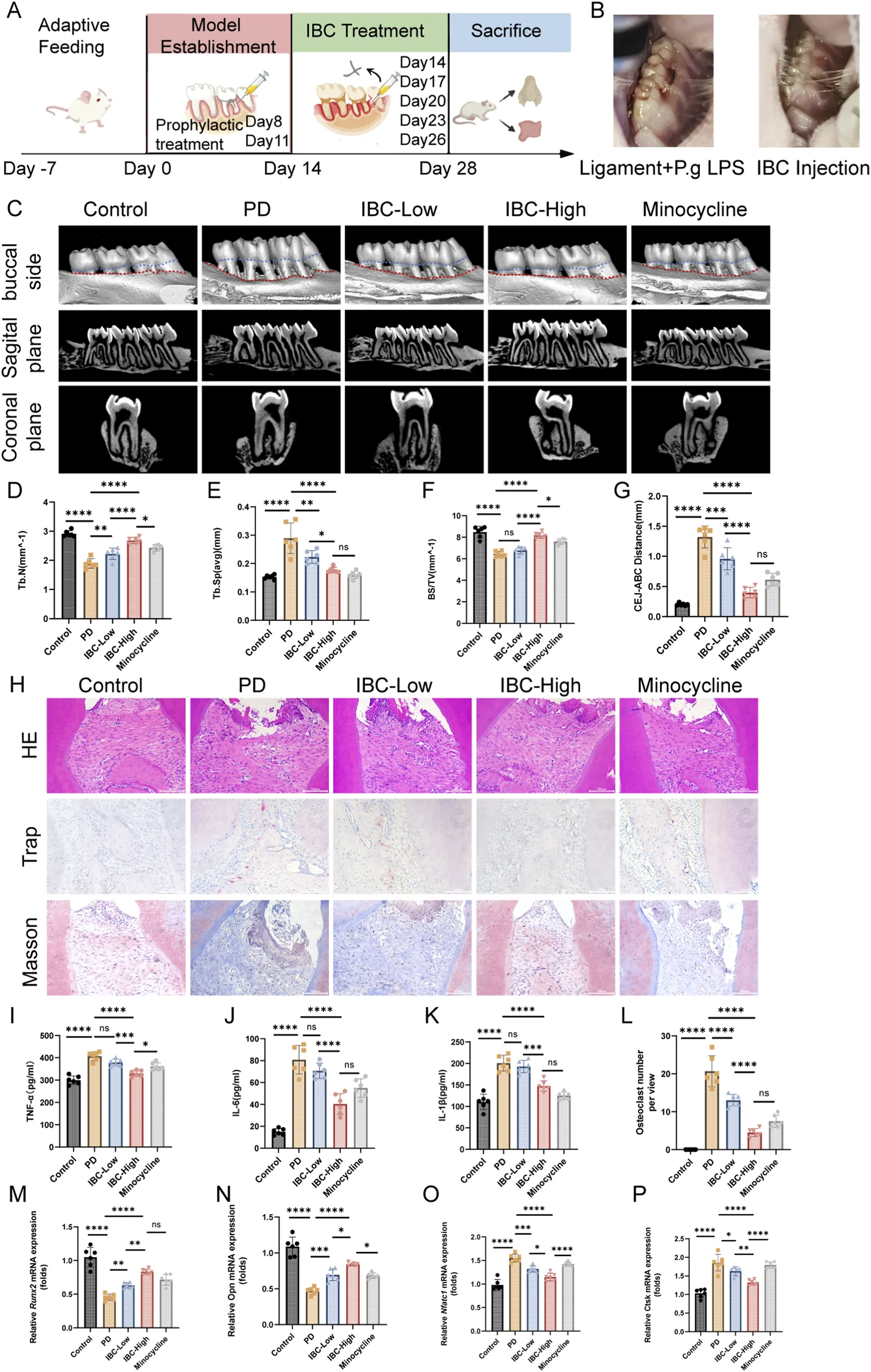
IBC regulates alveolar bone homeostasis and inflammation in experimental periodontitis. **(A)** Schematic timeline of the experimental design and IBC administration. **(B)** Representative intraoral photographs of the murine gingival status. **(C)** Images of the micro-syringe used for subgingival injection. **(D–H)** Representative 3D Micro-CT reconstructions of maxillary molars and quantitative morphometric analysis of Tb.N, Tb. Sp, BS/TV, and CEJ-ABC distance. **(I, M)** Representative images of H&E, TRAP, and Masson’s trichrome staining in periodontal tissues with corresponding quantification of TRAP-positive osteoclasts. **(J–L)** Concentration of pro-inflammatory cytokines (TNF-α, IL-6, IL-1β) in gingival tissues measured by ELISA. **(M–P)** Quantitative real-time PCR (qRT-PCR) analysis of osteogenic marker transcripts (*Runx2*, *Opn*) and osteoclastogenic marker transcripts (*Nfatc1*, *Ctsk*) in rat gingival tissues. Data are presented as mean ± SD (n = 6 per group). Scale bar = 100 μm *p < 0.05, **p < 0.01, ***p < 0.001, ****p < 0.0001.

### IBC orchestrates macrophage M2 polarization and quenches ROS-mediated ferroptosis *in vivo*

Systemic oxidative stress is a primary trigger of periodontal immune destruction. Visualized by DHE staining, the PD-induced overwhelming production of reactive oxygen species (ROS) in gingival tissues was profoundly quenched following IBC administration (Figures 3A,D). Driven by this ameliorated oxidative microenvironment, we evaluated the corresponding macrophage polarization. Immunofluorescence analysis showed that IBC markedly suppressed the expression of the M1 marker (iNOS) while upregulating the M2 marker (CD206) within CD68^+^ macrophages (Figures 3B,C,E,F), indicating a robust M1-to- M2 phenotypic switch. Consistently, qRT-PCR analysis showed that IBC increased Arg1 expression and reduced iNos expression in gingival tissues (Figures 3G, H).

**FIGURE 3:**
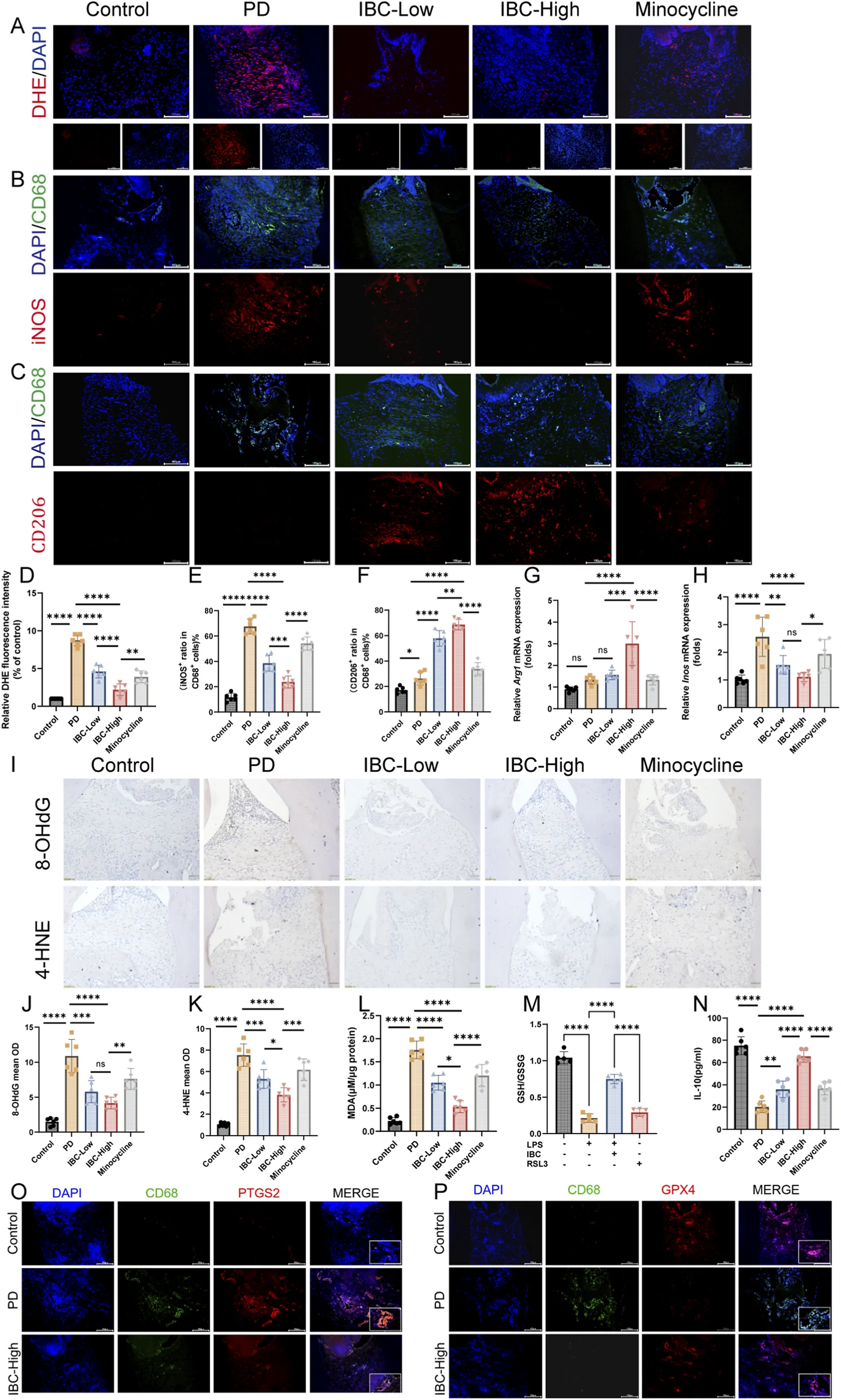
IBC modulates macrophage polarization and oxidative status *in vivo*. **(A,D)** Representative images and quantitative fluorescence intensity of dihydroethidium (DHE) staining in periodontal tissues. **(B,C,E,F)** Immunofluorescence co-staining of CD68 (green) with iNOS (red) or CD206 (red), and proportional analysis of positive cells. **(G,H)** Relative mRNA expression levels of Arg1 and iNos in gingival tissues. **(I–K)** Representative immunohistochemical images and quantitative optical density (OD) analysis of 8-OHdG and 4-HNE. **(L, M)** Biochemical assessment of malondialdehyde (MDA) content and the GSH/GSSG ratio. **(N)** IL-10 levels in gingival tissues measured by ELISA. **(O)** Immunofluorescence co-localization analysis of the pan-macrophage marker CD68 (green) and the core target PTGS2 (red) in periodontal sections. **(P)** Immunofluorescence co-localization analysis of CD68 (green) and the anti-ferroptotic protein GPX4 (red) in periodontal sections. Data are presented as mean ± SD (n = 6 per group). Scale bar = 100 μm *p < 0.05, **p < 0.01, ***p < 0.001, ****p < 0.0001.

Constant ROS is closely linked to macrophage ferroptosis. Because of this, we also checked for local lipid peroxidation and DNA damage markers. Immunohistochemistry revealed that IBC notably attenuated the massive accumulation of 8-OHdG and 4-HNE in diseased periodontal tissues (Figures 3I–K). In parallel, IBC reduced local malondialdehyde (MDA) levels and restored the protective GSH/GSSG ratio (Figures 3L,M). Functional cytokine profiling revealed a significant elevation in the secretion of the anti-inflammatory cytokine IL-10 *in vivo* (Figure 3N), indicative of successful ferroptosis evasion and an M2 phenotypic transition. To explicitly prove that this anti-ferroptosis effect occurred exactly inside the macrophages, we performed an *in vivo* immunofluorescence analysis. As demonstrated in Figures 3O,P and quantitatively confirmed by area fraction and mean fluorescence intensity (MFI) measurements (Supplementary Figure S1I,J), IBC treatment effectively reversed the high levels of PTGS2 caused by the disease. Concurrently, it restored GPX4 expression specifically within CD68^+^ macrophages. Furthermore, we evaluated the local expression of mitochondrial iron transporters, revealing that IBC effectively downregulated the iron importer Mfrn1 and upregulated the exporter Cisd1 in the periodontal microenvironment (Supplementary Figure S1G,H). This localized regulation confirms that IBC directly helps these cells avoid ferroptosis and switch to the reparative M2 phenotype.

These *in vivo* findings collectively demonstrated that IBC alleviated oxidative stress, suppressed ferroptosis within macrophages, and promoted M2 polarization, thereby contributing to the resolution of periodontal inflammation.

### Integrative target prediction nominates PTGS2 as a candidate mediator of IBC action

To dissect the molecular mechanisms underlying the therapeutic effects of IBC, a multi-omics network pharmacology approach was employed. By intersecting the predicted targets of IBC with PD-associated disease genes across multiple databases, we identified a robust subset of 81 overlapping targets (Figure 4A). Following the construction of a protein-protein interaction (PPI) network, the top 25% of genes were sequentially screened using three topological analysis algorithms (cytoNCA, MCODE, and cytoHubba). Strikingly, subsequent Venn diagram intersection analysis revealed that all three scoring methods uniquely converged on a single paramount core target: PTGS2 (Figures 4B–F).

**FIGURE 4:**
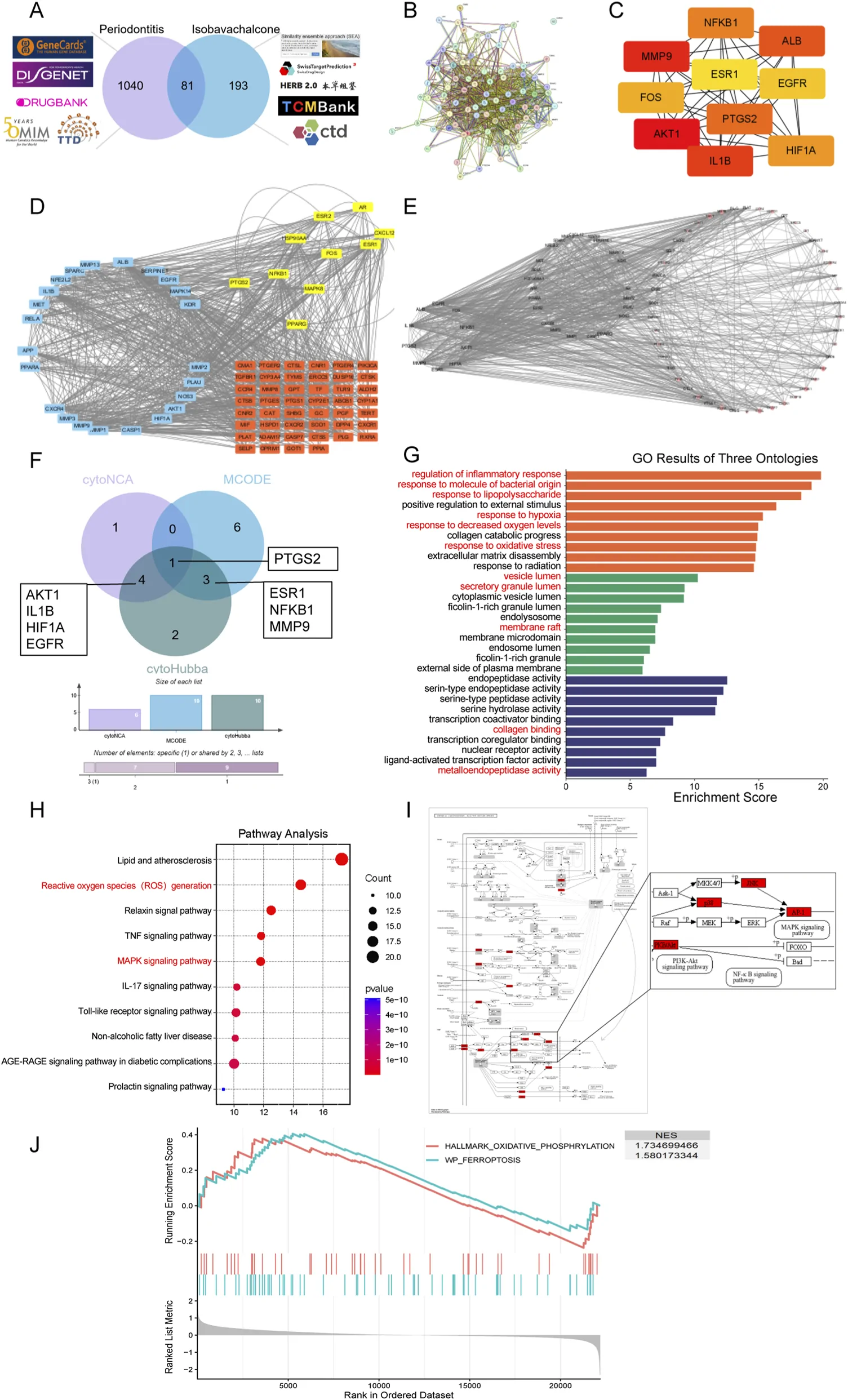
Multi-omics screening and network pharmacology analysis to identify core therapeutic targets of IBC. **(A)** Venn diagram showing the intersection of IBC-predicted targets and periodontitis-associated genes. **(B)** Protein–protein interaction (PPI) network constructed from the intersecting genes. **(C–E)** Top 25% of hub genes identified by topological analysis using three distinct scoring algorithms: CytoNCA, MCODE, and CytoHubba. **(F)** Core target PTGS2 pinpointed by intersecting the hub genes obtained from the three algorithms. **(G,H)** GO and KEGG analyses categorizing major biological functions and downstream signaling pathways. **(I)** Mapping of core overlapping targets onto the classical KEGG pathway diagram for “Reactive oxygen species (ROS) generation”. Specific downstream components, including the MAPK cascade (p38 and JNK), are highlighted in red to indicate their roles within the signaling network. **(J)** GSEA plots showing enrichment of oxidative phosphorylation and ferroptosis signatures in the GSE10334 dataset.

Subsequent GO and KEGG enrichment analyses demonstrated that these overlapping targets were highly enriched in pathways governing “Reactive oxygen species (ROS) generation,” “Lipid and atherosclerosis,” and the “MAPK signaling pathway” et al. (Figures 4G,H). To further illustrate the specific gene-to-term associations, cnetplots were constructed for the Biological Process (BP), Cellular Component (CC), and Molecular Function (MF) categories, detailing the complex interaction networks between hub genes and these enriched functions (Supplementary Figure S2A-C). Crucially, by directly mapping our core targets onto the classical KEGG pathway diagram for ROS generation, we explicitly pinpointed the MAPK cascade—specifically highlighting JNK and p38—as the pivotal downstream effectors mediating this oxidative stress (Figure 4I). To further corroborate these overarching functional trends, Gene Set Enrichment Analysis (GSEA) was performed utilizing the GEO dataset GSE10334. The results demonstrated a profound positive enrichment of signatures related to “oxidative phosphorylation” and “ferroptosis” (Figure 4J). Collectively, these bioinformatic cues strongly suggested that IBC exerted its anti-ferroptotic function by engaging these crucial inflammatory and oxidative nodes.

### Molecular dynamics and CETSA physically confirm the stable engagement of IBC with PTGS2

Based on the multi-omics prediction, we subjected the core targets to molecular docking. Interestingly, IBC displayed the highest binding affinity with PTGS2 (Binding energy: −9.3 kcal/mol, Table 1; Figure 5A). To further characterize ferroptosis-related alterations *in vivo*, we examined a panel of classical ferroptosis-associated markers. The PD group showed a pro-ferroptotic transcriptional profile, characterized by increased expression of Tfrc, Acsl4, Hmox1, and Alox5, together with reduced expression of *F*th1, Gpx4, Slc7a11, and Nrf2. Notably, Gpx4 exhibited the most pronounced reduction among the anti-ferroptotic markers, whereas IBC treatment markedly restored Gpx4 expression and partially reversed the ferroptosis-associated transcriptional imbalance (Figure 5B). To validate this direct physical interaction, Molecular Dynamics (MD) simulations were performed. The trajectory analyses, including RMSD, RMSF, and Rg, demonstrated a highly stable dynamic behavior of the IBC-PTGS2 complex over the 100 ns simulation (Figures 6A–D). Free energy landscapes (FEL) and MM-GBSA calculations further confirmed a favorable thermodynamic profile driven by robust hydrogen bonding and hydrophobic interactions (Figures 6E–I). Crucially, CETSA experiments structurally validated that IBC binding significantly enhanced the thermal stability of the PTGS2 protein compared to the control (Figures 6J,K), physically locking PTGS2 as the direct pharmacological target of IBC.

**TABLE 1:** Molecular docking parameters of Isobavachalcone (IBC) with core hub targets associated with periodontitis.

Gene symbol	Protein full name	PDB ID	Binding energy (kcal/mol)
PTGS2	Prostaglandin G/H synthase 2	3HS5	−9.3
MMP9	Matrix metalloproteinase-9	1ITV	−8.3
ESR1	Estrogen receptor 1	9BQ1	−8.1
AKT1	AKT serine/threonine kinase 1	5WBL	−8
IL1B	Interleukin-1 beta	7Z9K	−7
NFκB1	Nuclear factor kappa B subunit 1	1U36	−6.7
HIF1A	Hypoxia inducible factor 1 subunit alpha	8HE0	−7.3
EGFR	Epidermal growth factor receptor	2XKN	−7.7

**FIGURE 5:**
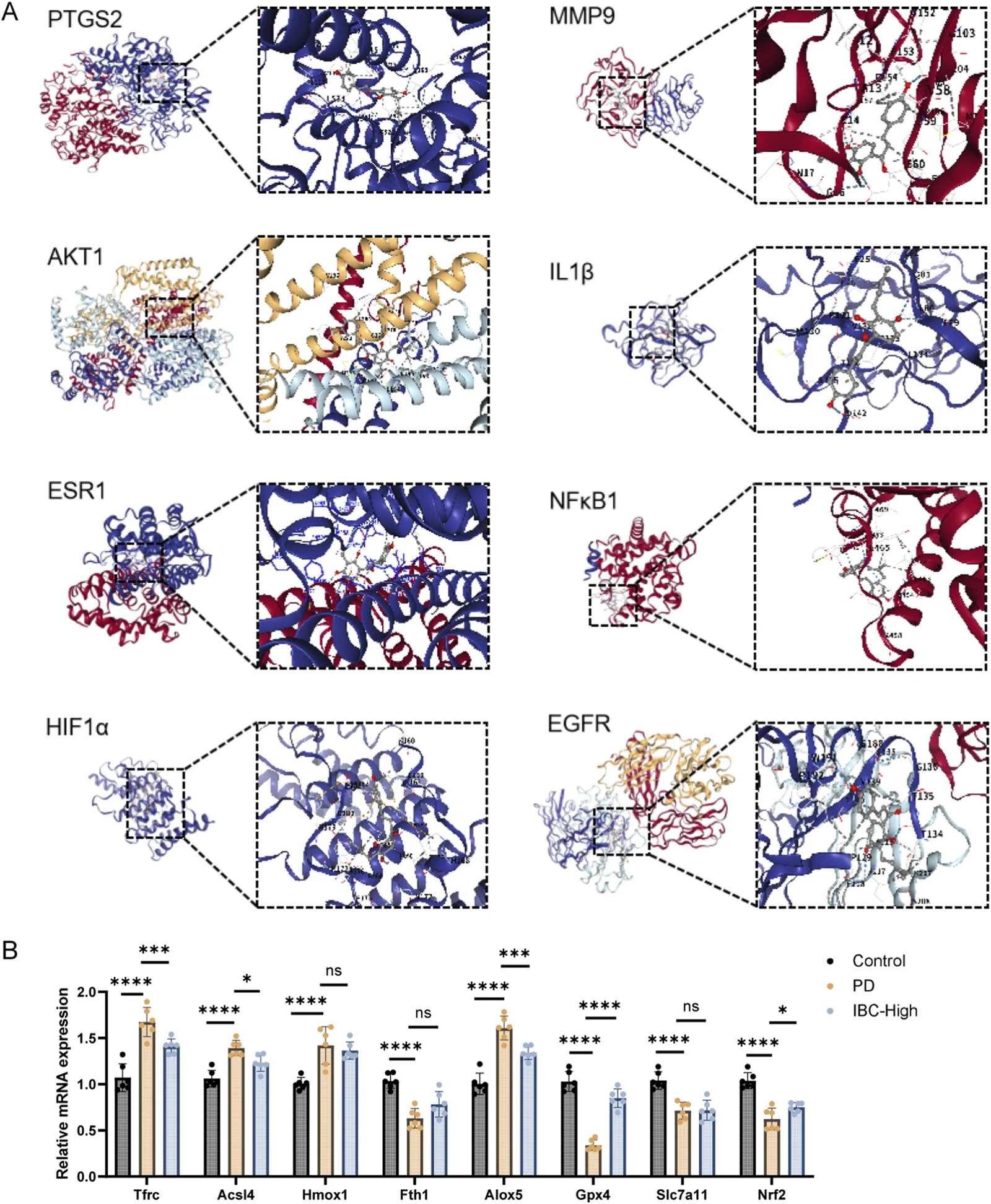
Molecular docking and transcriptional validation of potential targets. **(A)** 3D molecular docking models showing binding poses between IBC and candidate hub targets. **(B)** Relative mRNA expression of iron metabolism and ferroptosis-associated genes. Data are presented as mean ± SD (n = 6 per group). *p < 0.05, **p < 0.01, ***p < 0.001, ****p < 0.0001.

**FIGURE 6:**
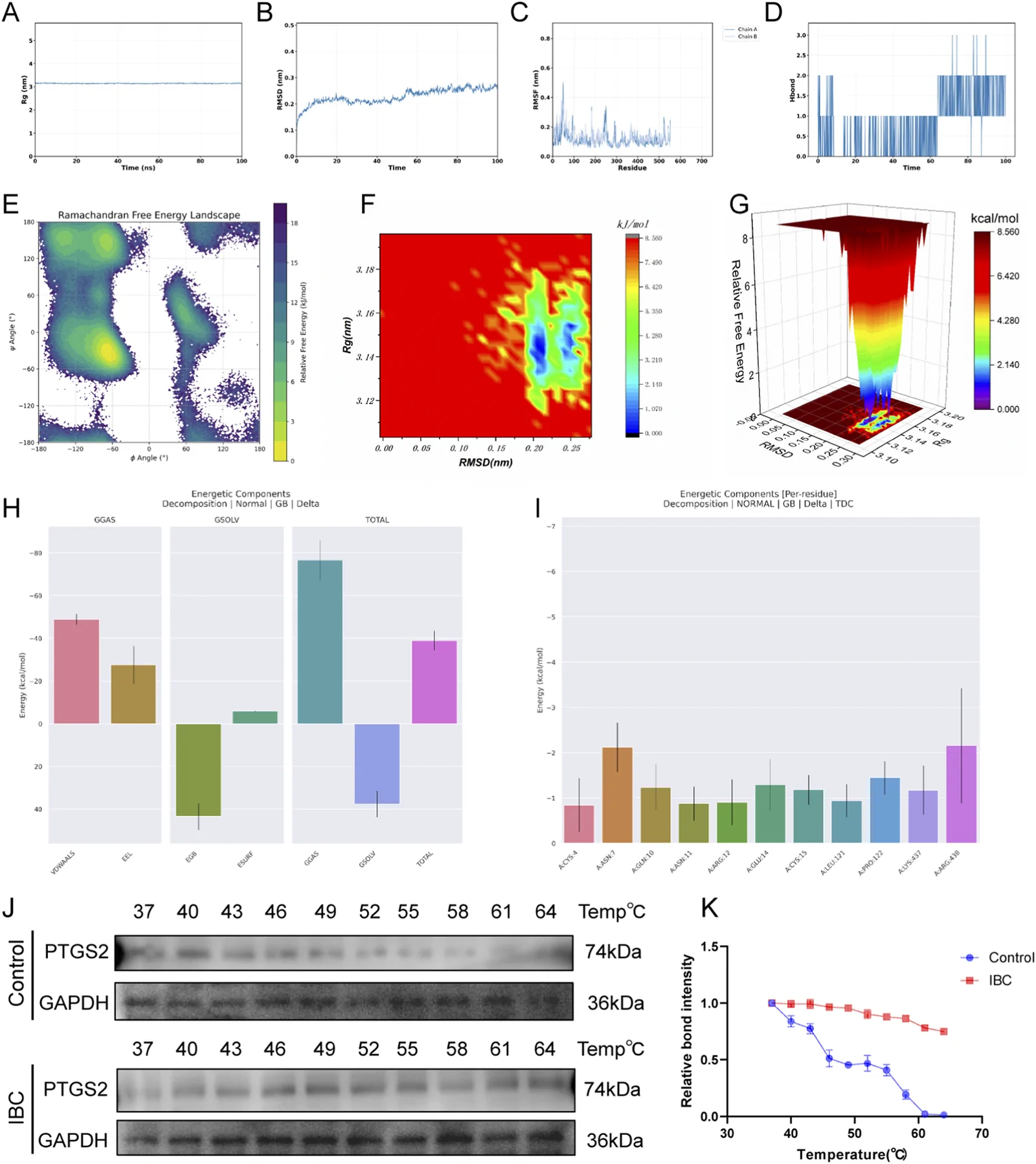
Molecular dynamics simulations and CETSA physically confirm the stable engagement of IBC with PTGS2. **(A)** Trajectory analysis of the IBC-PTGS2 complex over 100 ns of molecular dynamics simulation: radius of gyration (Rg). **(B)** Trajectory analysis: root mean square deviation (RMSD). **(C)** Trajectory analysis: root mean square fluctuation (RMSF). **(D)** Trajectory analysis: number of intermolecular hydrogen bonds. **(E)** Ramachandran free energy landscape characterizing the conformational states. **(F)** RMSD-Rg free energy landscape (FEL) characterizing the conformational states. **(G)** Three-dimensional representation of relative free energy. **(H)** Binding free energy decomposition (MM-GBSA). **(I)** Per-residue energetic contribution analysis. **(J)** Cellular thermal shift assay (CETSA) followed by Western blotting to assess thermal stability of PTGS2. **(K)** Densitometric quantification of CETSA-associated PTGS2 thermal stability (n = 3 independent experiments).

To further evaluate whether GPX4, a key ferroptosis-protective protein, could represent an alternative direct target of IBC, comparative molecular docking and CETSA analyses were performed. Molecular docking revealed that IBC exhibited a substantially weaker predicted interaction with GPX4 (binding energy: −4.2 kcal/mol) compared with PTGS2 (−9.3 kcal/mol) (Supplementary Figure S5A,B). In addition, CETSA analysis showed no significant thermal stabilization of GPX4 following IBC treatment, in contrast to the marked thermal shift observed for PTGS2 (Supplementary Figure S5C,D). These findings suggest that GPX4 is unlikely to represent the predominant direct binding target of IBC and further support PTGS2 as the major pharmacological target involved in IBC-mediated ferroptosis regulation.

To further exclude potential interference from other cellular proteins or environmental factors, we performed microscale thermophoresis (MST) using purified recombinant human PTGS2 protein in a cell-free system. As shown in Supplementary Figure S8A-C, IBC bound directly to PTGS2 in a dose-dependent manner, with a dissociation constant (Kd) of 0.012 μM, as determined from three independent biological replicates. This nanomolar-range Kd indicates a strong and direct molecular interaction between IBC and PTGS2. Together with the CETSA results, these data provide robust evidence that PTGS2 is a direct and high-affinity binding target of IBC. Having established that IBC directly binds to PTGS2 with high affinity, we next investigated whether this direct interaction translates into functional inhibition of PTGS2 enzymatic activity. To this end, RAW264.7 cells were treated with control, LPS, LPS + IBC (36 μM), or LPS + celecoxib (36 μM, a selective COX-2 inhibitor used as a positive control), and COX activity in the cell lysate supernatant was measured using a COX activity fluorescence assay kit. As shown in Supplementary Figure S8D, LPS stimulation markedly increased COX-2 activity compared with the control group, confirming the induction of PTGS2 catalytic function under inflammatory conditions. Treatment with IBC significantly attenuated the LPS-induced elevation of COX-2 activity, with an inhibitory effect comparable to that of celecoxib at the same concentration. These results demonstrate that IBC not only binds to PTGS2 directly but also functionally suppresses its enzymatic activity, reinforcing the conclusion that PTGS2 is a relevant functional target of IBC in the context of periodontitis.

To further determine whether the IBC-induced reduction in PTGS2 protein expression was attributable to transcriptional downregulation or to direct effects on PTGS2 protein stability/activity, we measured PTGS2 mRNA levels by qPCR in control, LPS, and LPS + IBC groups. As shown in Supplementary Figure S8I, LPS stimulation markedly increased PTGS2 mRNA expression compared with the control group, confirming transcriptional activation of PTGS2 under inflammatory conditions. However, IBC treatment only slightly reduced LPS-induced PTGS2 mRNA levels, and the effect was not statistically significant. In contrast, PTGS2 protein expression was substantially decreased by IBC treatment, as shown by Western blotting. These results suggest that the inhibitory effect of IBC on PTGS2 is unlikely to be mediated primarily through transcriptional suppression. Rather, IBC appears to act mainly at the protein level, consistent with the direct binding and enzymatic inhibition demonstrated by the MST and COX activity assays.

### Single-cell and clinical evidence substantiate PTGS2-associated macrophage ferroptosis in human periodontitis

To bridge the gap between our *in vivo* findings and human pathology, we systematically mapped the expression distribution of the core therapeutic targets within the human periodontal microenvironment using the scRNA-seq dataset (GSE171213). As illustrated by the UMAP clustering and feature density plots (Figures 7A–C), the majority of the overlapping core targets—most notably PTGS2, IL1B, MMP9, HIF1A, and NFKB1—were predominantly enriched within innate immune cell populations, specifically peaking in the neutrophil and monocytic/macrophage clusters. While neutrophils serve as the acute first responders during periodontal infection, macrophages act as the central orchestrators of the persistent chronic inflammatory response and subsequent alveolar bone destruction. Notably, our key target PTGS2 was highly expressed in both macrophages and neutrophils. Given that periodontitis is a chronic infectious microenvironment, we focused on macrophages for further investigation and selected the RAW264.7 cell line as our *in vitro* model. We subsequently validated this in human clinical gingival specimens. The demographic and periodontal clinical characteristics of enrolled participants, including age, sex distribution, probing depth (PD), clinical attachment loss (CAL), and bleeding index (BI), are summarized in Table 2. No significant differences were observed in age or sex distribution between healthy controls and patients with periodontitis, whereas the periodontitis group exhibited significantly increased PD, CAL, and BI values, confirming the clinical characteristics of periodontal tissue destruction. Immunohistochemistry and immunofluorescence verified an overwhelming surge of PTGS2 colocalized within CD68^+^ macrophages in PD patients, which inversely correlated with the profound depletion of the anti-ferroptotic defender GPX4 (Figures 7D–F). Western blot analysis of clinical tissues confirmed the hyperactivation of the MAPK pathway (p-JNK, p-p38) alongside PTGS2 upregulation (Figures 7G–N), cementing the clinical relevance of the PTGS2-MAPK-ferroptosis axis in PD.

**FIGURE 7:**
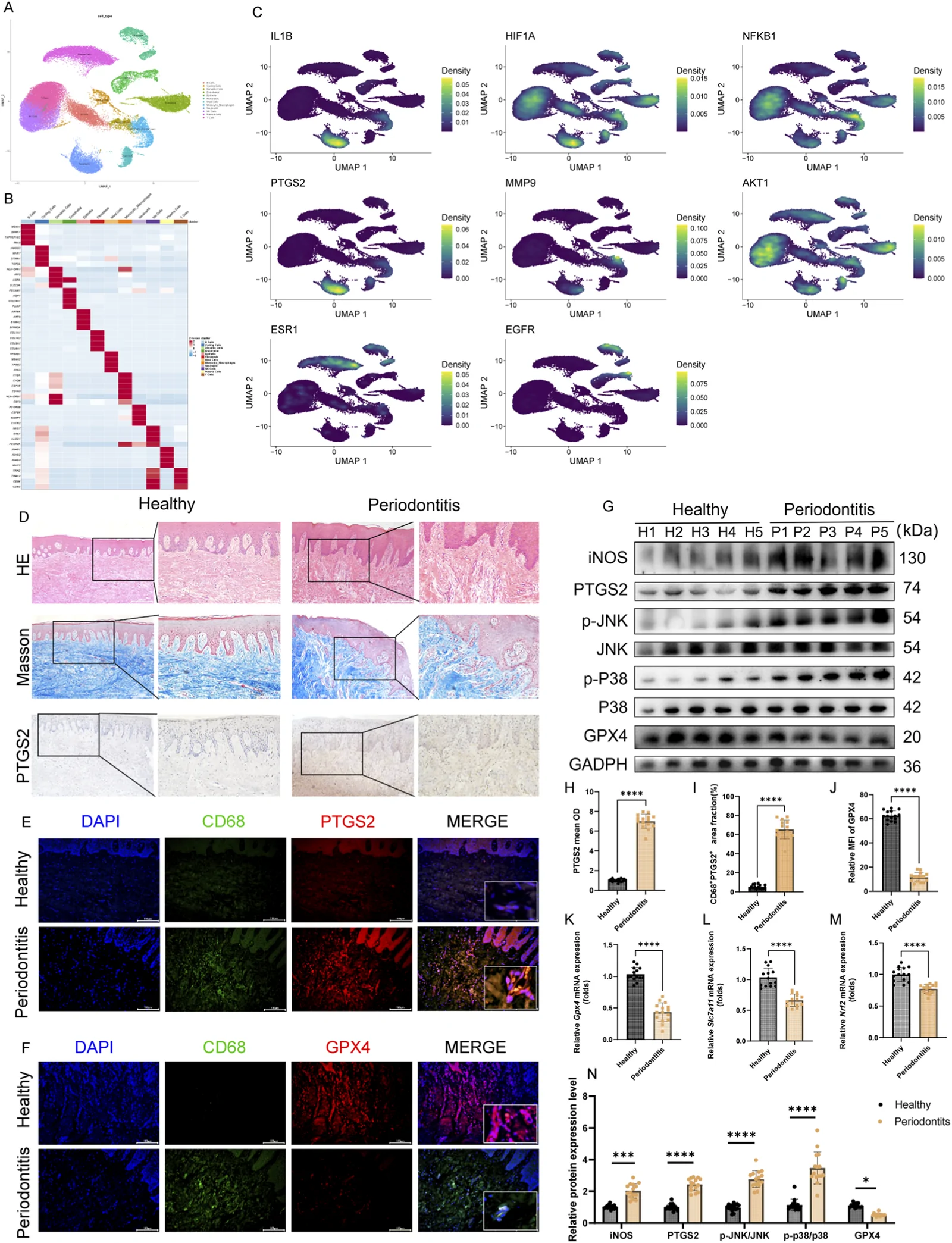
Single-cell and clinical evidence substantiate PTGS2-associated macrophage ferroptosis in human periodontitis. **(A)** UMAP plot illustrating major cell clusters from the scRNA-seq dataset (GSE171213) of gingival tissues. **(B)** Heatmap showing marker gene expression across distinct cell clusters. **(C)** UMAP feature plots detailing the expression distribution of core targets, highlighting the specific enrichment of PTGS2 in the macrophage subpopulation. **(D)** Representative H&E, Masson’s trichrome, and immunohistochemical staining of PTGS2 in clinical gingival samples. **(E,F)** Immunofluorescence double staining of CD68/PTGS2 and CD68/GPX4 in human clinical samples. **(G)** Evaluation of iNOS, PTGS2, MAPK cascade members (including phosphorylated and total JNK and p38), and GPX4 levels in human periodontal samples via Western blotting. **(H)** Quantification of the IHC staining results shown in panel **(D)**. **(I,J)** Quantitative analyses of the immunofluorescence staining results shown in panels E and F, respectively. **(K–M)** Relative mRNA expression levels of ferroptosis-related markers detected by qRT-PCR. **(N)** Densitometric quantification of the Western blot results shown in panel **(G)**. Data are presented as mean ± SD (n = 15 clinical samples per group). Scale bar = 100 μm *p < 0.05, **p < 0.01, ***p < 0.001, ****p < 0.0001.

**TABLE 2:** Demographic and clinical characteristics of the study participants.

Characteristics	Healthy (n = 15)	Periodontitis (n = 15)	P-value
Age (years)	42.3 ± 5.6	44.1 ± 6.2	>0.05
Sex (male/Female)	8/7	9/6	>0.05
Smoker (n, %)	0 (0%)	0 (0%)	-
Probing depth (mm)	1.84 ± 0.42	5.68 ± 0.85	<0.001 ***
Clinical attachment loss (mm)	0.00 ± 0.00	4.92 ± 1.15	<0.001 ***
Bleeding index (score 1–5)	1.2 ± 0.4	4.1 ± 0.6	<0.001 ***

Data are presented as mean ± SD, for continuous variables and number (percentage) for categorical variables. Statistical differences between the Healthy Control and Periodontitis groups were evaluated using Student’s t-test (for continuous variables like Age, PD, CAL, and BOP) or Chi-square test (for categorical variables like Gender and Smoking status). ***P < 0.001 indicates a statistically significant difference compared to the healthy control group. Abbreviations: PD, probing depth; CAL, clinical attachment loss; BOP, bleeding on probing.

### IBC facilitates M2 polarization by abrogating GPX4-dependent ferroptosis in macrophages *in vitro*

Cytotoxicity assays (CCK-8) revealed that treatment with 36 μM IBC for 24 h did not significantly affect cell viability, whereas viability decreased at 48 h. At 72 μM, IBC reduced viability at both 24 h and 48 h. Therefore, we selected 36 μM IBC with 24 h treatment for subsequent experiments to avoid confounding cytotoxic effects (Figure 8A). To simulate the inflammatory microenvironment *in vitro*, we challenged RAW 264.7 macrophages with LPS. To determine whether the immunomodulatory effects of IBC depend on the canonical anti-ferroptotic defense system, we employed a pharmacological rescue strategy using RSL3, a potent ferroptosis inducer.

**FIGURE 8:**
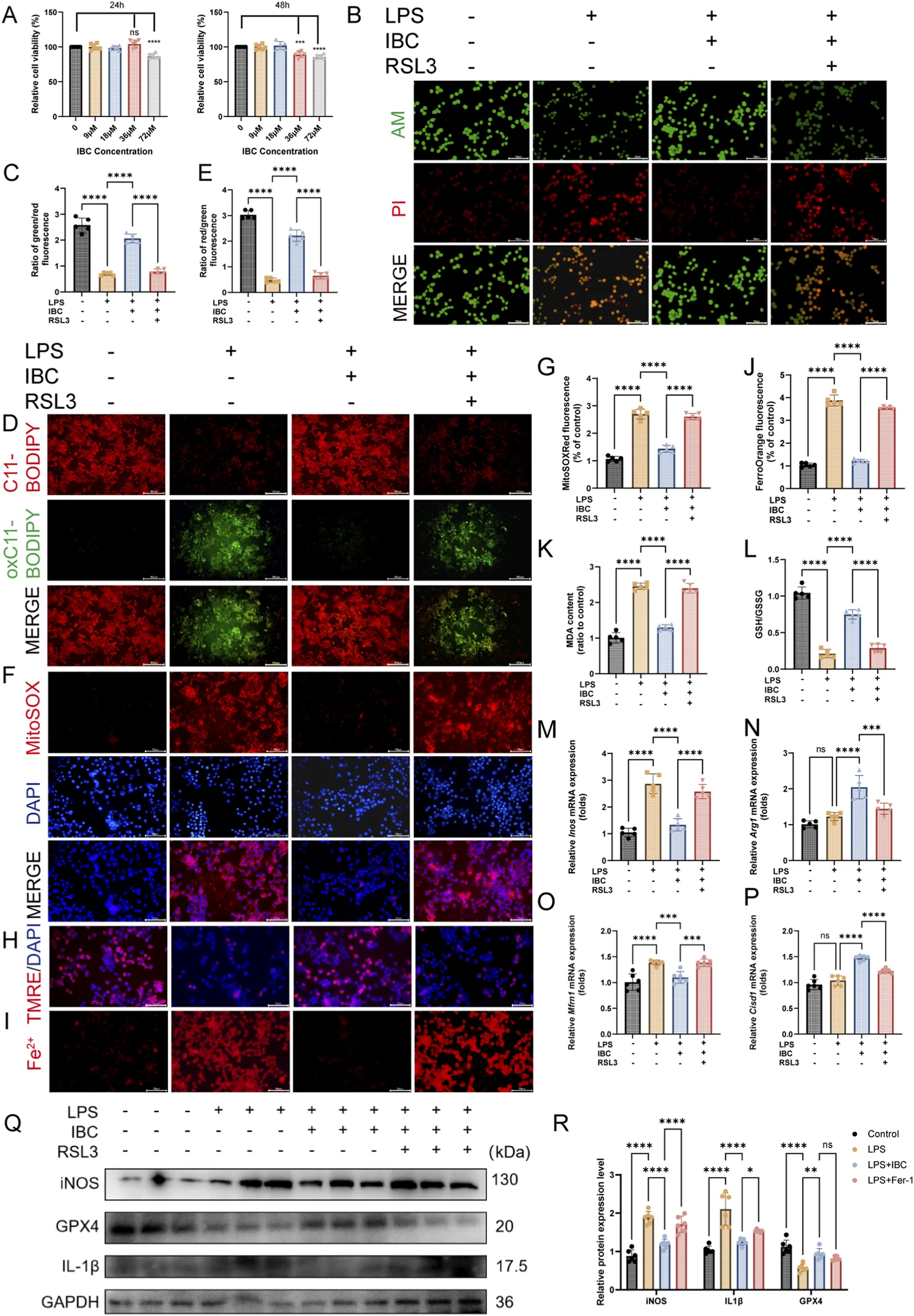
IBC regulates macrophage M2 polarization by suppressing GPX4-dependent ferroptosis *in vitro*. **(A)** IBC’s effect on RAW 264.7 cell viability at 24 and 48 h. **(B,C)** Cell survival analysis via Calcein-AM/PI staining and the corresponding green/red fluorescence ratio quantification. **(D,E)** Evaluation of lipid peroxidation using the C11-BODIPY probe and the related ratio of oxidized to reduced signals. **(F,G)** Detection of mitochondrial ROS through MitoSOX staining with the quantified fluorescence intensity. **(H)** Representative immunofluorescence images of TMRE staining (red) used to monitor mitochondrial membrane potential (Δψ_m_) in macrophages. **(I,J)** Visualization of intracellular iron (Fe^2+^) using FerroOrange and the related fluorescence measurement. **(K,L)** Biochemical quantification of intracellular MDA content and the GSH/GSSG ratio. **(M,N)** Transcriptional analysis of M1 (Inos) and M2 (Arg1) markers via qRT-PCR. **(O,P)** Transcriptional analysis of mitochondrial iron importer (Mfrn1) and exporter (Cisd1) mRNA levels in macrophages following LPS challenge and IBC treatment, with or without the addition of the GPX4 inhibitor RSL3. **(Q,R)** Representative Western blot bands and densitometric protein quantification for iNOS, GPX4, and IL-1β. Data are mean ± SD from three independent biological replicates (n = 6). Scale bar = 50 μm *p < 0.05, **p < 0.01, ***p < 0.001, ****p < 0.0001.

As expected, IBC treatment effectively reversed the LPS-induced loss of cell membrane integrity, as evidenced by the restoration of Calcein-AM signals and corresponding quantification (Figures 8B,C). This protection was accompanied by a marked decrease in lipid peroxidation, as shown by oxidized C11-BODIPY fluorescence (Figures 8D,E). Furthermore, IBC quenched mitochondrial superoxide overproduction, demonstrated by MitoSOX staining (Figures 8F,G). Crucially, IBC preserved the mitochondrial membrane potential (Δψm), as observed through the recovery of TMRE fluorescence and its relative quantitative analysis (Figure 8H; Supplementary Figure S3E), and concurrently prevented the accumulation of intracellular ferrous iron (Fe^2+^), indicated by FerroOrange staining and relative fluorescence measurement (Figures 8I,J). Biochemically, this comprehensive anti-ferroptotic effect was supported by decreased malondialdehyde (MDA) levels (Figure 8K) and restored GSH/GSSG ratios (Figure 8L).

We next investigated whether this ferroptotic status influenced subsequent macrophage polarization. Transcriptional analyses confirmed that IBC reduced the expression of the M1 marker *iNos* (Figure 8M) and promoted the M2 marker Arg*1* (Figure 8N). Moreover, IBC corrected the LPS-induced dysregulation of mitochondrial iron transporters by suppressing the iron influx transporter *Mfrn1* (Figure 8O) and upregulating the efflux transporter *Cisd1* (Figure 8P). Finally, Western blot analysis and densitometric quantification verified that IBC suppressed M1-associated inflammatory proteins (iNOS, IL-1β) while restoring the anti-ferroptotic defender GPX4 (Figures 8Q,R).

Strikingly, the concurrent administration of RSL3 completely abolished all these protective and immunomodulatory effects of IBC. Upon downstream GPX4 inhibition, the macrophages lost their ability to maintain mitochondrial iron homeostasis, as evidenced by the re-dysregulation of Mfrn1 and Cisd1 transcripts (Supplementary Figure S3A,B), and reverted to a severe state of lipid peroxidation and oxidative stress, thereby remaining arrested in a pro-inflammatory M1 state even in the presence of IBC. Collectively, these *in vitro* results demonstrated that IBC drove M2 polarization specifically by stabilizing the GPX4-dependent antioxidant axis and preserving mitochondrial fitness.

To further clarify whether the macrophage reparative phenotype induced by IBC was solely secondary to the alleviation of inflammatory and ferroptotic stress, or whether IBC itself possesses a direct macrophage-regulatory capacity, we treated resting RAW264.7 macrophages with IBC alone in the absence of LPS stimulation or ferroptosis induction. Compared with untreated macrophages, IBC treatment significantly increased the expression of the M2-associated marker Arg1 at the transcriptional level and enhanced IL-10 secretion in culture supernatants (Supplementary Figure S6). These findings indicate that IBC possesses an intrinsic capacity to prime macrophages toward a reparative phenotype under basal conditions.

Nevertheless, under pathological inflammatory conditions, the protective effects of IBC were further enhanced through suppression of ferroptotic stress and restoration of macrophage redox homeostasis, suggesting that both direct phenotype priming and indirect microenvironmental remodeling contribute to IBC-mediated macrophage regulation.

### IBC mitigates macrophage ferroptosis via the PTGS2-MAPK-ROS-GPX4 axis

Finally, we unraveled the hierarchical signaling cascade. IBC dose-dependently inhibited PTGS2 expression, which consecutively blunted the phosphorylation of JNK and p38 MAPK, whilst restoring GPX4 levels (Figures 9A–G). To confirm the pathological relevance of this axis *in vivo*, we examined the gingival tissues from rat models of experimental periodontitis. Consistent with our *in vitro* findings, Western blot analysis revealed that IBC treatment significantly suppressed the expression of iNOS and PTGS2, and inhibited the hyperactivation of the MAPK signaling proteins (p-JNK and p-p38) within the diseased periodontal microenvironment.

**FIGURE 9:**
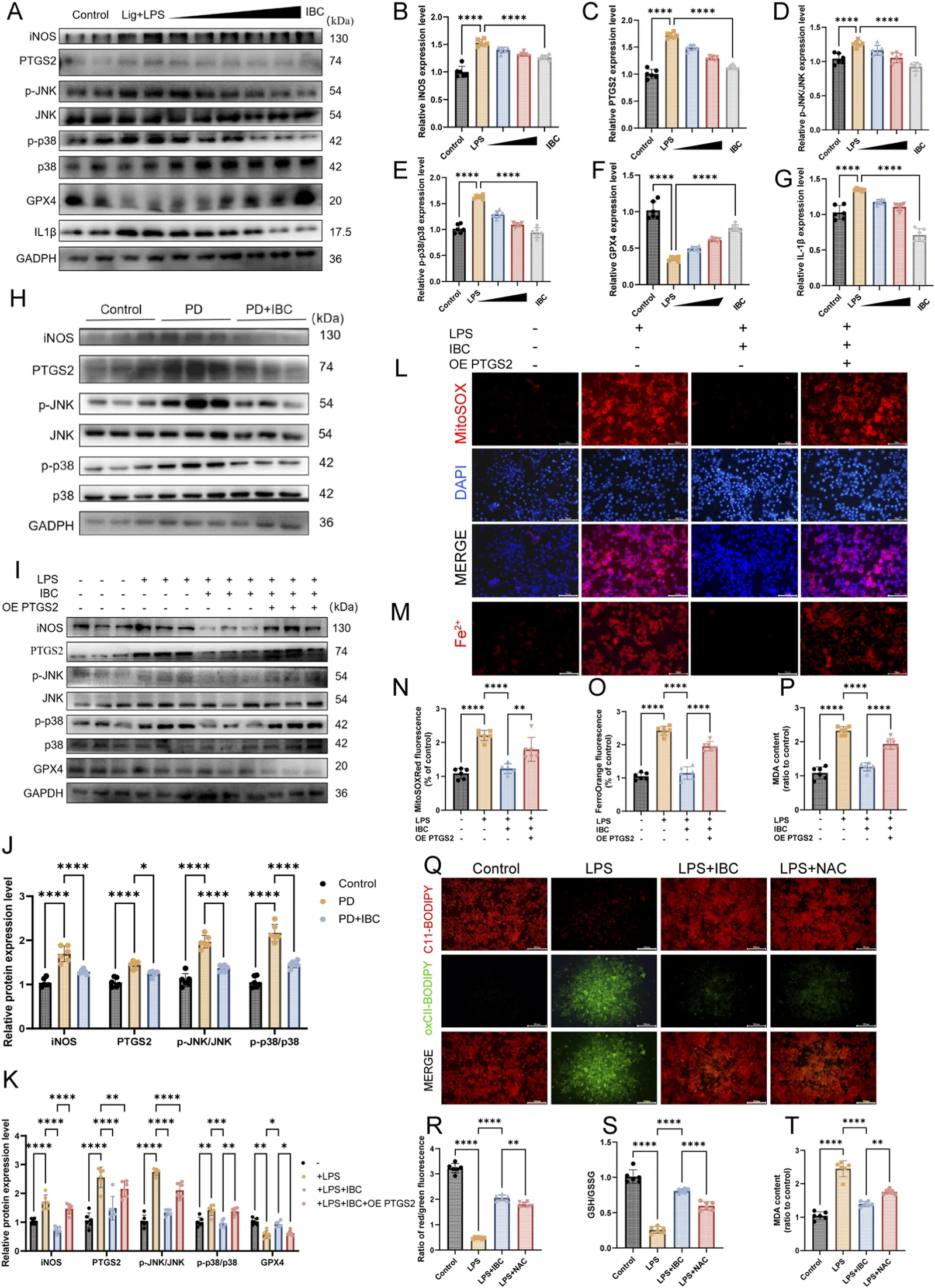
IBC mitigates macrophage ferroptosis via the PTGS2-MAPK-ROS-GPX4 axis. **(A–G)** Representative Western blots and quantitative analysis of iNOS, PTGS2, p-JNK, JNK, p-p38, p38, GPX4, and IL-1β in LPS-stimulated RAW264.7 cells treated with IBC in a dose-dependent manner. **(H, J)** Detection and quantification of iNOS, PTGS2, MAPK signaling proteins, and GPX4 in gingival tissues collected from rat models of experimental periodontitis. **(I, K)** Representative Western blots and quantitative analysis of the PTGS2-MAPK-GPX4 signaling axis in macrophages following OE-PTGS2 plasmid transfection and IBC treatment to assess pathway rescue. **(L–P)** Microscopic visualization of MitoSOX and FerroOrange signals, accompanied by the measurement of fluorescence intensity and MDA levels in cells undergoing OE-PTGS2 rescue treatments. **(Q–T)** Representative C11-BODIPY fluorescence imaging, GSH/GSSG ratio, and MDA content assessment in macrophages treated with the ROS scavenger NAC. Data Note: Data are presented as mean ± SD from three independent biological replicates (n = 6) for *in vitro* experiments, and n = 6 per group for *in vivo* analysis. Scale bar = 50 μm.

To definitively establish whether PTGS2 is the indispensable hub for IBC’s action, we conducted functional rescue experiments by overexpressing PTGS2 (OE PTGS2) in macrophages. Strikingly, PTGS2 overexpression almost completely abrogated the beneficial effects of IBC. Western blot analysis confirmed that OE PTGS2 reactivated the downstream MAPK signaling (p-JNK, p-p38) and severely depleted GPX4 expression despite the presence of IBC (Figures 9H–K). Consequently, the levels of mitochondrial superoxide (MitoSOX), intracellular ferrous iron (Fe^2+^, FerroOrange), and MDA surged back to pathological levels (Figures 9L–P). In parallel, OE-PTGS2 abrogated IBC’s regulation on mitochondrial iron transport, reverting Mfrn1 and Cisd1 mRNA expression to disease-like states (Supplementary Figure S3C,D), and severely compromised the mitochondrial membrane potential (Δψm) (Supplementary Figure S3F).

We used a specific tool called NAC to clear ROS. This helped us confirm that ROS buildup happens after the PTGS2-MAPK axis and is the main cause of GPX4-dependent ferroptosis. Co-treatment with NAC flawlessly mimicked the antioxidant effects of IBC, effectively abolishing LPS-induced lipid peroxidation (C11-BODIPY), restoring the GSH/GSSG ratio, decreasing MDA accumulation, and rescuing GPX4 expression (Figures 9Q–T). Collectively, these definitive genetic (OE PTGS2) and pharmacological (NAC) rescue data confirmed that IBC preserved mitochondrial function and quenched ferroptosis strictly by suppressing the PTGS2-MAPK-ROS amplification loop. To further determine the hierarchical position of JNK/p38 MAPK activation in the PTGS2-associated signaling axis, we employed selective inhibitors of JNK (SP600125) and p38 (SB203580). RAW264.7 cells were stimulated with LPS in the presence or absence of these inhibitors, and the expression of PTGS2 and GPX4 was measured by qPCR, while mitochondrial ROS production was assessed by MitoSOX Red staining. As shown in Supplementary Figure S8E-H, neither SP600125 nor SB203580 significantly affected LPS-induced PTGS2 mRNA expression, indicating that MAPK inhibition does not act upstream of PTGS2. In contrast, treatment with either inhibitor significantly attenuated LPS-induced mitochondrial ROS accumulation and restored GPX4 mRNA levels. These data demonstrate that JNK/p38 activation functions downstream of PTGS2 and upstream of ROS production and GPX4 downregulation, providing further support for the proposed PTGS2–JNK/p38–ROS–GPX4 signaling axis in IBC-mediated protection against ferroptosis.

To further determine whether PTGS2 activation itself contributes to ferroptotic regulation, RAW264.7 macrophages were transfected with an OE-PTGS2 plasmid under basal conditions without inflammatory stimulation, ferroptosis induction, or IBC treatment. Compared with cells transfected with the control vector, PTGS2 overexpression markedly increased lipid peroxidation, as indicated by elevated oxidized C11-BODIPY fluorescence and increased MDA levels. Moreover, PTGS2 overexpression resulted in enhanced intracellular ferrous iron accumulation, reflected by increased FerroOrange fluorescence, together with reduced GPX4 protein expression (Supplementary Figure S4).

=These results demonstrate that PTGS2 activation alone is sufficient to induce ferroptosis-associated oxidative injury, iron dysregulation, and loss of antioxidant defense in macrophages, further supporting the role of PTGS2 as an upstream inflammatory regulator of macrophage ferroptotic susceptibility.

To further evaluate the pharmacological relevance of PTGS2 as a functional target of IBC, we compared the effects of IBC with those of celecoxib, a well-established selective COX-2 inhibitor, in LPS-stimulated RAW264.7 cells. Consistent with the COX-2 activity assay described above, both IBC (36 μM) and celecoxib (36 μM) significantly inhibited LPS-induced COX-2 activity, with comparable efficacy (Supplementary Figure S8D). Western blot analysis further showed that IBC and celecoxib similarly suppressed LPS-induced PTGS2 protein expression (Supplementary Figure S8J), indicating that the inhibitory effects of IBC on PTGS2 are comparable to those of the reference inhibitor at both the enzymatic activity and protein levels.

We next asked whether pharmacological inhibition of PTGS2 by celecoxib could phenocopy the anti-ferroptotic effects of IBC. Treatment with either IBC or celecoxib markedly reduced intracellular ferrous iron accumulation, as detected by FerroOrange staining, decreased MDA levels, and restored the GSH/GSSG ratio (Supplementary Figure S8K-M). These results demonstrate that selective COX-2 inhibition with celecoxib mimics the protective effects of IBC against LPS-induced ferroptotic changes, further supporting that PTGS2 inhibition is a major contributor to the anti-inflammatory and anti-ferroptotic activity of IBC in this model.

#### Mechanistic insights into IBC-mediated periodontal protection

Based on our integrated multi-omics screening, molecular docking, MD simulations, CETSA, and *in vivo* and *in vitro* functional rescue experiments, we propose a mechanistic model for IBC in alleviating periodontitis (Figure 10). Under inflammatory conditions, IBC physically binds to and antagonizes PTGS2, specifically in macrophages. This direct inhibition disrupts the downstream JNK/p38 MAPK phosphorylation cascade, thereby interrupting the ROS amplification loop. In parallel, IBC prevents mitochondrial iron overload by downregulating the iron influx transporter MFRN1 and upregulating the efflux transporter CISD1, and suppresses Fenton reactions, thereby restoring the GPX4/GSH antioxidant system. This coordinated blockade of the ferroptotic pathway promotes the polarization of macrophages toward an anti-inflammatory M2 phenotype, which is essential for limiting tissue damage and initiating the natural repair of periodontal bone structures.

**FIGURE 10:**
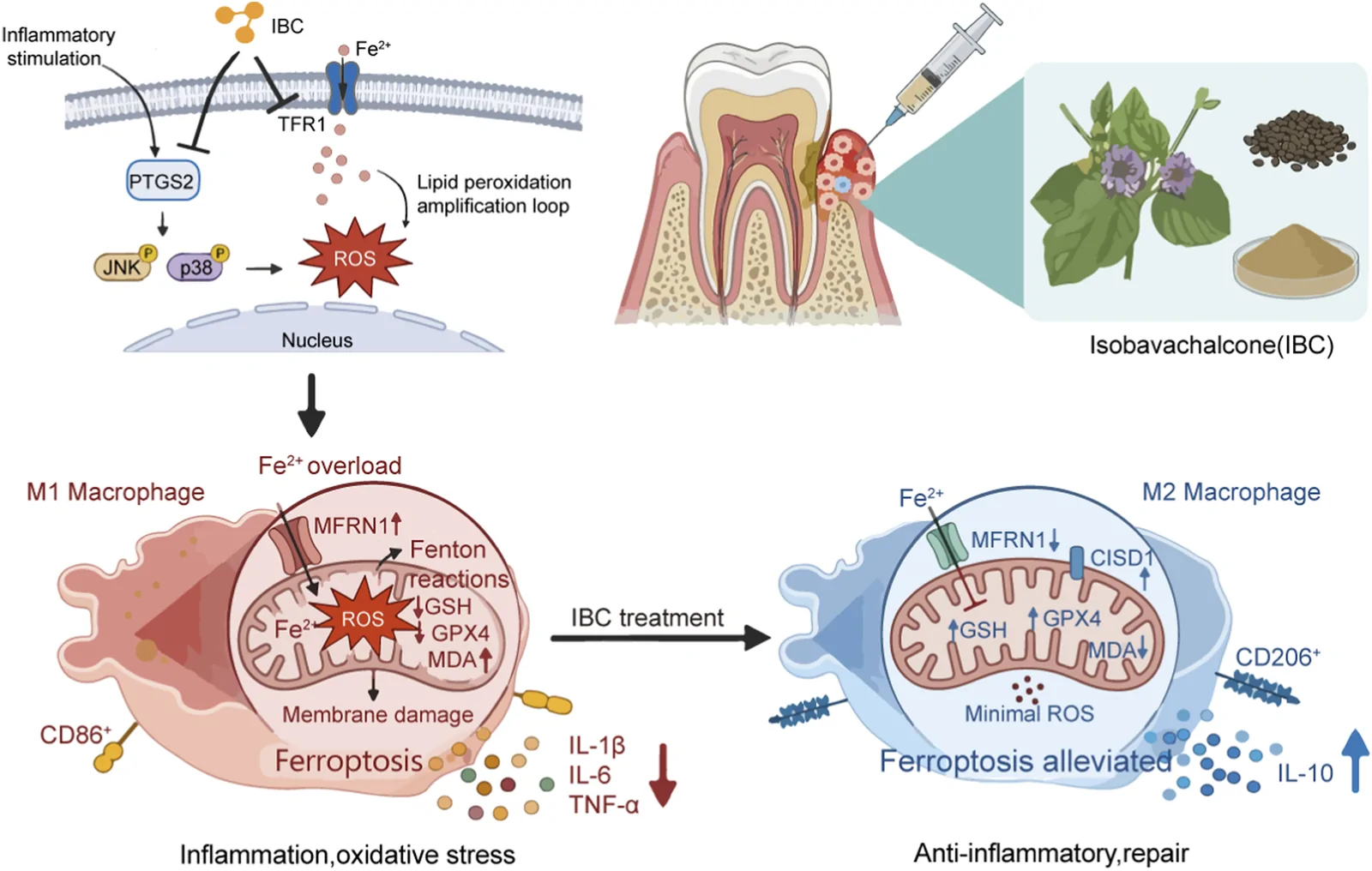
Graphical Abstract: Proposed mechanism of IBC in alleviating periodontitis. Schematic illustration of the anti-ferroptotic and pro-M2 polarization effects of IBC in macrophages. IBC directly targets PTGS2, disrupting the JNK/p38 MAPK-ROS amplification loop, regulating mitochondrial iron transporters (MFRN1/CISD1), and restoring the GPX4/GSH antioxidant system, ultimately promoting M2 polarization and periodontal tissue repair.

## Discussion

Periodontitis has become a major oral health challenge worldwide because of its high prevalence, chronic progression, and close association with tooth loss, masticatory dysfunction, and impaired quality of life (Darveau, 2010; Tonetti et al., 2018). Although mechanical plaque control, scaling and root planing, and adjunctive antimicrobial strategies remain the cornerstone of periodontal therapy, these approaches mainly reduce the microbial burden and are often insufficient to fully correct the dysregulated immune microenvironment that drives persistent inflammation and alveolar bone destruction (Shapouri-Moghaddam et al., 2018; Garaicoa-Pazmino et al., 2019). Moreover, repeated antibiotic use may raise concerns regarding antimicrobial resistance, disruption of local microbial ecology, and limited long-term efficacy (Hajishengallis, 2014; Lamont et al., 2018). Therefore, identifying host-modulatory agents capable of simultaneously controlling inflammation, oxidative injury, and tissue-destructive immune responses represents an important direction for improving periodontal therapy. In this context, plant-derived natural compounds have attracted increasing interest because many of them possess relatively low toxicity, broad biological activity, and multi-target regulatory potential. IBC, a naturally occurring prenylated chalcone, has been reported to exert protective effects in several inflammatory and degenerative disease models, including experimental colitis, rheumatoid arthritis, osteoporosis, and neuroinflammatory disorders (Wang X. et al., 2021; Zhou et al., 2021b; Wang et al., 2022). However, its therapeutic value in periodontal inflammation and the underlying immunopharmacological mechanisms have not been fully defined. In the present study, we systematically evaluated the protective effects of IBC in experimental periodontitis and further explored its regulatory actions on macrophage inflammatory phenotypes, ferroptosis, and redox homeostasis. To strengthen the translational relevance of these findings, we further integrated human scRNA-seq analysis and gingival specimens from patients with periodontitis, which confirmed the enrichment of PTGS2 in periodontal innate immune/macrophage populations and revealed increased CD68/PTGS2 signals, reduced macrophage-associated GPX4, and activation of JNK/p38 MAPK signaling in diseased gingival tissues. Our findings demonstrate that IBC attenuates periodontal inflammation and alveolar bone loss, suppresses macrophage ferroptosis, promotes a shift from M1-like inflammatory activation toward M2-like reparative polarization, and restores GPX4-dependent antioxidant defense. Mechanistically, these effects are associated with inhibition of the PTGS2-linked MAPK–ROS signaling cascade, suggesting that IBC may act as a potential host-modulatory small molecule for controlling macrophage-driven periodontal tissue destruction.

Macrophages are indispensable innate immune regulators in periodontal tissues and participate in both host defense and tissue remodeling. During the early phase of microbial challenge, macrophage activation contributes to pathogen clearance, phagocytosis of cellular debris, and initiation of inflammatory defense (Hajishengallis and Chavakis, 2021; Zhao J. et al., 2022). However, when periodontal inflammation becomes persistent, sustained exposure to bacterial components, inflammatory cytokines, and oxidative stress can drive macrophages toward a predominantly M1-like pro-inflammatory state. This phenotype is characterized by increased production of iNOS, TNF-α, IL-1β, IL-6, and other inflammatory mediators, which further amplify local immune activation, promote osteoclastogenesis, and aggravate periodontal connective tissue and bone destruction (Yuan et al., 2022; Cao et al., 2025). In contrast, M2-like macrophages are generally associated with inflammation resolution, efferocytosis, extracellular matrix remodeling, and tissue repair through the secretion of anti-inflammatory and pro-regenerative mediators such as IL-10, Arg1, and CD206 (Chen X. et al., 2022; Zhang et al., 2024). Therefore, the balance between M1-like inflammatory activation and M2-like reparative polarization is a critical determinant of whether the periodontal immune microenvironment progresses toward destructive inflammation or returns to tissue homeostasis. In the present study, experimental periodontitis induced a macrophage phenotype characterized by enhanced M1-associated inflammatory activation and impaired reparative polarization, whereas IBC treatment reduced pro-inflammatory macrophage markers and cytokine production while increasing M2-associated markers. Importantly, additional experiments under basal conditions showed that IBC alone increased Arg1 expression and IL-10 secretion in macrophages, suggesting that IBC may possess an intrinsic capacity to prime macrophages toward a reparative phenotype. However, the enhanced effects observed under inflammatory conditions indicate that IBC-mediated macrophage remodeling is likely achieved through both direct phenotype regulation and indirect correction of the oxidative and ferroptotic microenvironment. These findings suggest that IBC does not merely suppress inflammation in a nonspecific manner, but rather reprograms macrophage immune status toward a reparative phenotype. Such macrophage rebalance may represent an important mechanism by which IBC limits periodontal inflammation, reduces alveolar bone loss, and facilitates the restoration of periodontal immune homeostasis.

Ferroptosis has recently emerged as an important form of regulated cell death linking oxidative stress, iron dyshomeostasis, lipid peroxidation, and inflammatory tissue injury (Dixon and Olzmann, 2024; Zheng and Conrad, 2025). Unlike apoptosis or necroptosis, ferroptosis is mainly driven by iron-dependent accumulation of lipid peroxides and the failure of antioxidant defense systems, particularly the cystine–GSH–GPX4 axis. In the periodontal inflammatory microenvironment, persistent microbial stimulation and excessive ROS production may render macrophages highly vulnerable to ferroptotic stress (Wickert et al., 2024; Chen et al., 2025). Once ferroptosis occurs, damaged macrophages can further release inflammatory mediators, lipid peroxidation products, and danger-associated signals, thereby amplifying local inflammation and aggravating alveolar bone destruction. Therefore, macrophage ferroptosis may represent not only a consequence of oxidative injury but also an active contributor to unresolved periodontal inflammation (Yang et al., 2023; Feng et al., 2024). In this study, experimental periodontitis exhibited a typical pro-ferroptotic profile, including enhanced oxidative DNA and lipid damage, increased lipid peroxidation, disturbed iron-related gene expression, depletion of GSH-dependent antioxidant capacity, and marked suppression of GPX4. Consistently, *in vitro* LPS stimulation induced ferroptosis-like injury in macrophages, as evidenced by increased ROS/lipid ROS accumulation, mitochondrial dysfunction, iron overload, dysregulated expression of mitochondrial iron transport genes, and downregulation of GPX4-associated antioxidant defense. IBC treatment substantially reversed these pathological alterations, reduced oxidative and lipid peroxidation damage, restored GSH/GSSG balance, and preserved GPX4 expression, indicating that its protective effect is closely associated with the suppression of macrophage ferroptosis. Importantly, ferroptosis induction by RSL3 weakened the protective and pro-reparative effects of IBC, whereas antioxidant intervention partially mimicked or enhanced its action, further supporting the involvement of the ROS–GPX4 ferroptotic axis. These findings suggest that IBC-mediated inhibition of macrophage ferroptosis may help interrupt the vicious cycle between oxidative stress, inflammatory macrophage activation, and periodontal tissue destruction, thereby contributing to the restoration of periodontal immune and redox homeostasis.

PTGS2/COX-2 is traditionally recognized as an inducible inflammatory enzyme involved in prostaglandin synthesis, but accumulating evidence also links PTGS2 activation to oxidative stress, lipid peroxidation, and ferroptosis-associated inflammatory injury (Zhou et al., 2021a; Martín-Vázquez et al., 2023). In the present study, integrative target prediction first nominated PTGS2 as a candidate mediator connecting IBC pharmacological action with macrophage-driven periodontal inflammation. This candidate axis was further supported by human scRNA-seq analysis and patient gingival specimen validation, which showed PTGS2 enrichment in innate immune/macrophage clusters, increased PTGS2 expression in CD68^+^ macrophages, reduced macrophage-associated GPX4, and activation of JNK/p38 MAPK signaling in periodontitis tissues. Molecular docking and molecular dynamics simulations indicated that IBC could form a stable interaction with the PTGS2 protein, while CETSA further supported IBC–PTGS2 engagement in cells. Although PTGS2 has frequently been considered a ferroptosis-associated marker, our additional gain-of-function experiments demonstrated that PTGS2 overexpression alone induced lipid peroxidation, iron accumulation, and GPX4 depletion in macrophages under basal conditions. These findings suggest that PTGS2 may function not only as a marker associated with ferroptotic stress but also as an upstream inflammatory amplifier that increases macrophage susceptibility to ferroptotic injury. Mechanistically, PTGS2 activation in inflammatory macrophages was associated with increased JNK/p38 MAPK phosphorylation, ROS accumulation, mitochondrial iron dyshomeostasis, GPX4 suppression, and lipid peroxidation. Recent studies have highlighted that macrophage-derived ROS are not only cytotoxic byproducts but also redox signaling mediators that shape inflammatory pathway activation, while MAPK signaling, particularly JNK and p38, participates in macrophage inflammatory responses and can cooperate with oxidative stress to sustain inflammatory amplification (Canton et al., 2021; Li et al., 2023; Luecke et al., 2024; Zhao et al., 2024). Consistent with this MAPK–ROS inflammatory crosstalk, IBC suppressed PTGS2-associated JNK/p38 activation, reduced ROS and lipid peroxidation, restored GPX4-dependent antioxidant defense, and alleviated mitochondrial iron-related imbalance. Importantly, PTGS2 overexpression partially reversed these protective effects of IBC, reactivated JNK/p38 MAPK signaling, aggravated oxidative and ferroptotic stress, and weakened the shift toward a reparative macrophage phenotype. These rescue findings indicate that PTGS2 participates in IBC-mediated regulation of macrophage ferroptosis and immune polarization. Thus, the PTGS2–MAPK–ROS axis may function as an upstream inflammatory amplifier linking PTGS2 activation to ferroptotic macrophage injury, and its suppression represents a plausible mechanism by which IBC restores periodontal redox balance and immune homeostasis. Nevertheless, considering the pleiotropic pharmacological properties of IBC as a naturally occurring prenylated chalcone, PTGS2-independent mechanisms may also contribute to its overall antioxidant and anti-inflammatory effects. Previous studies have reported that IBC can modulate cellular redox homeostasis through mechanisms beyond PTGS2/COX-2 signaling. For example, IBC was shown to induce ROS-mediated apoptosis by interacting with the selenocysteine-containing antioxidant enzyme thioredoxin reductase 1 (TrxR1), leading to TrxR1 inhibition, ROS accumulation, and endoplasmic reticulum stress in prostate cancer cells (Li et al., 2018). In hepatocellular carcinoma cells, IBC was also reported to disrupt mitochondrial respiration and induce cytotoxicity through ROS accumulation and Akt suppression, suggesting that mitochondrial redox regulation may represent another context-dependent mechanism of IBC action (Zhao L. et al., 2022). In addition, IBC has been shown to exert anti-inflammatory effects through pathways such as NF-κB p65 inhibition in experimental colitis, ERK/NF-κB suppression and macrophage M1 polarization inhibition in osteoporosis models, and PI3K/AKT- and JAK/STAT-associated regulation in rheumatoid arthritis models (Wang M. et al., 2021; Zhou et al., 2021b; Wang et al., 2022). Therefore, although our PTGS2 overexpression rescue experiments, NAC intervention, and COX enzymatic activity validation support the involvement of the PTGS2-associated MAPK–ROS–GPX4 axis in IBC-mediated macrophage protection, we cannot completely exclude additional PTGS2-independent redox or inflammatory pathways. Future studies combining PTGS2 loss-of-function models, selective PTGS2/COX-2 pharmacological controls, unbiased redox proteomics, and pathway-specific inhibitors will be required to further distinguish the relative contribution of PTGS2-dependent and PTGS2-independent mechanisms in the therapeutic effects of IBC against periodontal inflammation.

Furthermore, our comparative studies with celecoxib, a clinically used selective COX-2 inhibitor, revealed that IBC suppressed PTGS2 enzymatic activity, reduced PTGS2 protein expression, and attenuated ferroptosis-related changes (including ferrous iron accumulation, MDA production, and GSH/GSSG imbalance) to a similar extent as celecoxib. These findings not only reinforce the functional relevance of PTGS2 as a major target of IBC but also highlight the therapeutic potential of IBC in the management of periodontitis. Given that chronic inflammation and oxidative stress are central drivers of periodontal tissue destruction, pharmacological inhibition of PTGS2 may represent a promising strategy to interrupt the PTGS2–MAPK–ROS–GPX4 axis and limit ferroptotic damage in the periodontium. Compared with synthetic COX-2 inhibitors, IBC is a naturally occurring prenylated chalcone with a favorable safety profile and pleiotropic anti-inflammatory and antioxidant activities, which may offer additional therapeutic benefits beyond PTGS2 inhibition. Nevertheless, further preclinical studies are warranted to evaluate the selectivity, pharmacokinetics, long-term safety, and *in vivo* efficacy of IBC before it can be considered as a candidate therapeutic agent for periodontitis.

Although consistent protective effects of IBC were observed in both experimental periodontitis models and macrophage-based *in vitro* systems, several limitations should be acknowledged. Given the pleiotropic antioxidant properties of natural polyphenolic compounds, we further evaluated whether GPX4 could represent an alternative direct target of IBC. Comparative molecular docking analysis showed that IBC exhibited a substantially weaker predicted interaction with GPX4 than with PTGS2 (−4.2 kcal/mol vs. −9.3 kcal/mol). Moreover, CETSA analysis demonstrated that IBC treatment did not induce an evident thermal stabilization shift of GPX4, in contrast to the significant thermal stabilization observed for PTGS2. These comparative results suggest that GPX4 is unlikely to represent the predominant direct pharmacological target of IBC, whereas PTGS2 may serve as the major target mediating the anti-ferroptotic effects of IBC. Nevertheless, considering the complex biological activities of natural polyphenolic compounds, we acknowledge that IBC may exert additional indirect regulatory effects on GPX4 expression or activity through downstream signaling pathways, and future biochemical studies will be required to further elucidate these potential secondary mechanisms. Second, PTGS2 overexpression rescue experiments provided functional support for the involvement of PTGS2 in IBC-mediated macrophage regulation; however, macrophage-specific Ptgs2 knockout models are still needed to confirm its *in vivo* contribution. In addition, although RAW264.7 macrophages provided a convenient and reproducible model for mechanistic investigations, further validation using primary macrophages, such as bone marrow-derived macrophages (BMDMs), and human-derived macrophage systems is warranted to confirm the physiological relevance and translational potential of these findings. Future studies should further evaluate the effects of IBC in more clinically relevant macrophage models, together with long-term local delivery strategies, pharmacokinetic characteristics, and biosafety profiles. In summary, this study demonstrates that IBC attenuates periodontal inflammation and alveolar bone destruction by suppressing macrophage ferroptosis and restoring macrophage immune balance. Mechanistically, IBC engages PTGS2 and restrains the PTGS2-associated JNK/p38 MAPK–ROS–GPX4 signaling axis, thereby reducing oxidative injury, lipid peroxidation, and ferroptotic macrophage activation while promoting a reparative M2-like phenotype. These findings deepen our understanding of macrophage ferroptosis as a druggable inflammatory mechanism in periodontitis and provide experimental support for the development of IBC as a potential host-modulatory small molecule for periodontal inflammatory destruction.

## Conclusion

In conclusion, as depicted in the schematic model (Figure 10), we demonstrate that isobavachalcone (IBC) directly targets PTGS2 and disrupts a JNK/p38 MAPK-dependent ROS amplification loop, while concurrently restoring mitochondrial iron homeostasis through the MFRN1/CISD1 axis, thereby preserving GPX4/GSH-mediated antioxidant defenses. Functionally, these effects suppress ferroptosis and promote macrophage polarization toward a reparative phenotype. Collectively, these findings elucidate a PTGS2-centered regulatory network linking inflammatory signaling to ferroptotic cell death and highlight IBC as a promising modulator of ferroptosis-associated inflammatory responses.

## Data Availability

The original contributions presented in the study are included in the article/Supplementary Material, further inquiries can be directed to the corresponding authors.
