# Supplementary material for: Isobavachalcone attenuates experimental periodontitis by suppressing PTGS2-associated macrophage ferroptosis and restoring periodontal immune homeostasis
Source: Front Pharmacol. 2026 Sep 21;17:1913548. doi: 10.3389/fphar.2026.1913548 (PMC13635172; doi:10.3389/fphar.2026.1913548)
Supplement: Supplementary file 1 [file Supplementaryfile1.docx]

**Supplementary Figure S1. IBC regulates the osteogenic/osteoclastogenic balance and mitochondrial iron homeostasis in vivo.** (A-F) Quantitative real-time PCR (qRT-PCR) analysis of osteogenic marker transcripts (*Runx2*, *Opn*, *Alp*) and osteoclastogenic marker transcripts (*Ctsk*, *Trap*, *Nfatc1*) in rat gingival tissues. (G-H) Transcriptional level assessment of mitochondrial iron importers (*Mfrn1*) and exporters (*Cisd1*) in the periodontal microenvironment via qRT-PCR. (I) Calculation of CD68+PTGS2+ area fractions based on the immunofluorescence co-localization analysis in periodontal sections (representative images are shown in Fig. 3O). (J) Measurement of relative mean fluorescence intensity (MFI) of GPX4 within the CD68+ macrophage population (representative images are shown in Fig. 3P). Scale bar = 100 μm. Data are presented as mean ± SD (n = 6 per group).

**Supplementary Figure S2. Integrated network visualization of Gene Ontology (GO) domains and KEGG pathway mapping.** (A–C) Cnetplots illustrating the specific gene-to-term associations within the three Gene Ontology (GO) categories: Biological Process (BP), Cellular Component (CC), and Molecular Function (MF). The dot size represents the number of genes enriched in each category.

**Supplementary Figure S3. Assessment of mitochondrial iron transport genes and mitochondrial membrane potential (Δψ_m_) under genetic and pharmacological rescue conditions.** (A–B) Quantitative real-time PCR (qRT-PCR) analysis of mitochondrial iron importer (Mfrn1) and exporter (Cisd1) mRNA levels in macrophages following LPS challenge and IBC treatment, with or without the addition of the GPX4 inhibitor RSL3.(C–D) Transcriptional level assessment of Mfrn1 and Cisd1 in macrophages subjected to LPS challenge and IBC treatment, with or without PTGS2 overexpression (OE PTGS2).(E) Quantitative analysis of relative TMRE fluorescence intensity corresponding to the experimental groups in fig8H. (F) Representative immunofluorescence images of TMRE staining (red) and the corresponding quantitative analysis of relative TMRE fluorescence intensity in macrophages treated with LPS and IBC, with or without PTGS2 overexpression (OE PTGS2). DAPI (blue) was used for nuclear counterstaining. Scale bar = 50 μm. Data are presented as mean ± SD of independent biological replicates. *p < 0.05, **p < 0.01, ***p < 0.001, ****p < 0.0001 (One-way ANOVA with Tukey's post hoc test).

**Supplementary Figure S4. PTGS2 overexpression induces ferroptosis-associated alterations in macrophages under basal conditions.** (A) Validation of PTGS2 overexpression by qRT-PCR. (B–C) Representative images and quantification of oxidized C11-BODIPY fluorescence indicating lipid peroxidation levels in RAW264.7 macrophages transfected with control vector or OE-PTGS2. (D) Quantification of intracellular malondialdehyde (MDA) levels. (E–F) Representative FerroOrange fluorescence images and quantification of intracellular ferrous iron levels. (G–H) Western blot analysis and densitometric quantification of GPX4 expression following PTGS2 overexpression. Data are presented as mean ± SD from three independent biological experiments. Statistical analysis was performed using unpaired Student’s t-test.

**Supplementary Figure S5. Comparative analysis of the interaction between IBC and GPX4.** (A) Predicted molecular docking model of the IBC–GPX4 complex. (B) Binding energy comparison showing the predicted interaction affinity between IBC and GPX4. (C–D) CETSA analysis of GPX4 thermal stability following IBC treatment. Representative Western blot images and melting curve quantification are shown.

**Supplementary Figure S6. IBC directly primes macrophages toward a reparative phenotype under basal conditions.** (A) Experimental design of RAW264.7 macrophages treated with IBC alone without LPS stimulation. (B) Relative mRNA expression of the M2-associated marker Arg1 following IBC treatment. (C) IL-10 secretion levels in culture supernatants measured by ELISA after IBC treatment. Data are presented as mean ± SD from three independent biological experiments. Statistical significance was determined using unpaired Student’s t-test.

**Supplementary Figure S7. Uncropped Western blot images corresponding to the main figures.** Full-length western blot images are provided for all immunoblots presented in the manuscript, including PTGS2, p-JNK, JNK, p-p38, p38, GPX4, iNOS, IL-1β, and GAPDH. Molecular weight markers and corresponding experimental groups are indicated.

**Supplementary Figure S8.Supplementary Figure S8. Additional target-engagement and functional validation of PTGS2/COX-associated signaling in IBC-mediated macrophage protection.** (A) Representative microscale thermophoresis (MST) traces showing the direct interaction between IBC and purified recombinant human PTGS2 protein. (B) MST dose–response binding curve generated from normalized fluorescence changes across serially diluted IBC concentrations. (C) Dissociation constant (Kd) values from three independent MST measurements, showing high-affinity binding between IBC and PTGS2. (D) COX enzymatic activity in RAW264.7 macrophages treated with Control, LPS, LPS + IBC, or LPS + Celecoxib. COX activity was measured using a fluorometric COX activity assay and normalized to total protein content. (E) Representative MitoSOX Red fluorescence images showing mitochondrial ROS levels in RAW264.7 macrophages treated with Control, LPS, LPS + SP600125, or LPS + SB203580. (F) Quantification of MitoSOX Red fluorescence intensity corresponding to panel E. (G–H) qRT-PCR analysis of Ptgs2 and Gpx4 mRNA expression in RAW264.7 macrophages treated with Control, LPS, LPS + SP600125, or LPS + SB203580. (I) qRT-PCR analysis of Ptgs2 mRNA expression in RAW264.7 macrophages treated with Control, LPS, IBC, or LPS + IBC. (J) Western blot analysis of PTGS2 protein expression in RAW264.7 macrophages treated with Control, LPS, LPS + IBC, or LPS + Celecoxib. (K) Representative FerroOrange fluorescence images showing intracellular ferrous iron accumulation in RAW264.7 macrophages treated with Control, LPS, LPS + IBC, or LPS + Celecoxib. (L) Quantification of intracellular malondialdehyde (MDA) levels. (M) Quantification of the GSH/GSSG ratio. Data are presented as mean ± SD from three independent experiments or six biological replicates, as indicated. Statistical significance was determined using one-way ANOVA followed by Tukey’s post hoc test. Scale bar = 50 μm. *p < 0.05, **p < 0.01, ***p < 0.001, ****p < 0.0001.

**Supplementary Table S1. Antibody information and dilution conditions.**

| **Target Protein** | **Host Species** | **Reactivity** | **Supplier** | **Catalog Number** | **Application & Dilution** |
| --- | --- | --- | --- | --- | --- |
| PTGS2 (COX-2) | Rabbit | H, M, R | Cell Signaling Technology | #12282 | WB (1:1000); IHC (1:200); IF (1:200) |
| Phospho-p38 MAPK (Thr180/Tyr182) | Rabbit | H, M, R | Cell Signaling Technology | #4511 | WB (1:1000) |
| p38 MAPK | Rabbit | H, M, R | Cell Signaling Technology | #8690 | WB (1:1000) |
| Phospho-SAPK/JNK (Thr183/Tyr185) | Rabbit | H, M, R | Cell Signaling Technology | #4668 | WB (1:1000) |
| SAPK/JNK | Rabbit | H, M, R | Cell Signaling Technology | #9252 | WB (1:1000) |
| GPX4 | Rabbit | H, M | Cell Signaling Technology | #52455 | WB (1:1000); IF (1:100) |
| iNOS | Rabbit | H, M, R | Cell Signaling Technology | #13120 | WB (1:1000); IF (1:200) |
| IL-1β | Rabbit | H, M, R | Cell Signaling Technology | #63124 | WB (1:1000) |
| GAPDH | Rabbit | H, M, R | Cell Signaling Technology | #2118 | WB (1:1000) |

**Supplementary Table S2. Primer sequences used for qRT-PCR.**

| Species | Gene symbol | Alias / full name | Forward primer (5′–3′) | Reverse primer (5′–3′) |
| --- | --- | --- | --- | --- |
| Human | GPX4 | Glutathione peroxidase 4 | GAGGCAAGACCGAAGTAAACTAC | CCGAACTGGTTACACGGGAA |
| Human | SLC7A11 | Solute carrier family 7 member 11 / xCT | GGTCCATTACCAGCTTTTGTACG | AATGTAGCGTCCAAATGCCAG |
| Human | NRF2 | NRF2 | CACATCCAGTCAGAAACCAGTGG | GGAATGTCTGCGCCAAAAGCTG |
| Human | GAPDH | Glyceraldehyde-3-phosphate dehydrogenase | GTCTCCTCTGACTTCAACAGCG | ACCACCCTGTTGCTGTAGCCAA |
| Rat | Runx2 | Runt-related transcription factor 2 | ATCCAGCCACCTTCACTTACACC | GGGACCATTGGGAACTGATAGG |
| Rat | Opn | Osteopontin / Opn | CTCGATGTCATCCCTGTGCC | TGATCAGAGGGCAGGAGTTC |
| Rat | Nfatc1 | Nuclear factor of activated T cells 1 | CACCGCATCACAGGGAAGAC | GCACAGTCAATGACGGCTC |
| Rat | Ctsk | Cathepsin K | CTCTGTGTCCATCGACTATCG | CAGAGTCAATGCCTCCGTTCT |
| Rat | Arg1 | Arginase 1 | CTGAAGTGGACAAGCTGGGAATT | CCTGAAAGTAGCCCTGTCTTGTA |
| Rat | Nos2 | Inducible nitric oxide synthase / iNos | CCTTGTTCAGCTACGCCTTC | GGCTTCAAGAGAGGAGGCTC |
| Rat | Gapdh | Glyceraldehyde-3-phosphate dehydrogenase | GAAGGTCGGTGTGAACGGAT | CCCATTTGATGTTAGCGGGAT |
| Mouse | Mrfn1 | Mitoferrin-1 / Mfrn1 | GCCACCCTACTCCACGATGCA | CAACTGAAGGCTGACTGGTGCT |
| Mouse | Cisd1 | CDGSH iron-sulfur domain-containing protein 1 | AAGACAACCCGAAGGTGGTGCA | CTTCGTTGTGCTTTATGTGAGCC |
| Mouse | Nos2 | Inducible nitric oxide synthase / iNos | GAGACAGGGAAGTCTGAAGCAC | CCAGCAGTAGTTGCTCCTCTTC |
| Mouse | Arg1 | Arginase 1 | CATTGGCTTGCGAGACGTAGAC | GCTGAAGGTCTCTTCCATCACC |
| Mouse | Ptgs2 | Prostaglandin-endoperoxide synthase 2 / Cox-2 | GCGACATACTCAAGCAGGAGCA | AGTGGTAACCGCTCAGGTGTTG |
| Mouse | Mmp9 | Matrix metallopeptidase 9 | GCTGACTACGATAAGGACGGCA | TAGTGGTGCAGGCAGAGTAGGA |
| Mouse | Akt1 | AKT serine/threonine kinase 1 | GGACTACTTGCACTCCGAGAAG | CATAGTGGCACCGTCCTTGATC |
| Mouse | Il1b | Interleukin 1 beta | TGGACCTTCCAGGATGAGGACA | GTTCATCTCGGAGCCTGTAGTG |
| Mouse | Esr1 | Estrogen receptor 1 | TCTGCCAAGGAGACTCGCTACT | GGTGCATTGGTTTGTAGCTGGAC |
| Mouse | Nfkb1 | Nuclear factor kappa B subunit 1 | GCTGCCAAAGAAGGACACGACA | GGCAGGCTATTGCTCATCACAG |
| Mouse | Hif1a | Hypoxia-inducible factor 1 alpha | CCTGCACTGAATCAAGAGGTTGC | CCATCAGAAGGACTTGCTGGCT |
| Mouse | Egfr | Epidermal growth factor receptor | GGACTGTGTCTCCTGCCAGAAT | GGCAGACATTCTGGATGGCACT |
| Mouse | Tfrc | Transferrin receptor 1 / Tfr1 | GAAGTCCAGTGTGGGAACAGGT | CAACCACTCAGTGGCACCAACA |
| Mouse | Acsl4 | Acyl-CoA synthetase long-chain family member 4 | CCTTTGGCTCATGTGCTGGAAC | GCCATAAGTGTGGGTTTCAGTAC |
| Mouse | Hmox1 | Heme oxygenase 1 | CACTCTGGAGATGACACCTGAG | GTGTTCCTCTGTCAGCATCACC |
| Mouse | Fth1 | Ferritin heavy chain 1 | GCCGAGAAACTGATGAAGCTGC | GCACACTCCATTGCATTCAGCC |
| Mouse | Alox5 | Arachidonate 5-lipoxygenase | TCTTCCTGGCACGACTTTGCTG | GCAGCCATTCAGGAACTGGTAG |
| Mouse | Gpx4 | Glutathione peroxidase 4 | CCTCTGCTGCAAGAGCCTCCC | CTTATCCAGGCAGACCATGTGC |
| Mouse | Slc7a11 | Solute carrier family 7 member 11 / xCT | CTTTGTTGCCCTCTCCTGCTTC | CAGAGGAGTGTGCTTGTGGACA |
| Mouse | Nfe2l2 | NRF2 | CAGCATAGAGCAGGACATGGAG | GAACAGCGGTAGTATCAGCCAG |
| Mouse | Gapdh | Glyceraldehyde-3-phosphate dehydrogenase | CATCACTGCCACCCAGAAGACTG | ATGCCAGTGAGCTTCCCGTTCAG |
